# Non-stationary phase of the MALA algorithm

**DOI:** 10.1007/s40072-018-0113-1

**Published:** 2018-04-17

**Authors:** Juan Kuntz, Michela Ottobre, Andrew M. Stuart

**Affiliations:** 1 Imperial College London, London, SW7 2AZ UK; 2 Mathematics Department, Heriot Watt University, Edinburgh, EH14 4AS UK; 3 Department of Computing and Mathematical Sciences, California Institute of Technology, Pasadena, CA 91125 USA

**Keywords:** Markov Chain Monte Carlo, Metropolis-Adjusted Langevin Algorithm, Diffusion limit, Optimal scaling, Primary 60J22, Secondary 60J20, 60H10

## Abstract

The Metropolis-Adjusted Langevin Algorithm (MALA) is a Markov Chain Monte Carlo method which creates a Markov chain reversible with respect to a given target distribution, $\pi ^N$, with Lebesgue density on ${\mathbb {R}}^N$; it can hence be used to approximately sample the target distribution. When the dimension *N* is large a key question is to determine the computational cost of the algorithm as a function of *N*. The measure of efficiency that we consider in this paper is the *expected squared jumping distance* (ESJD), introduced in Roberts et al. (Ann Appl Probab 7(1):110–120, 1997). To determine how the cost of the algorithm (in terms of ESJD) increases with dimension *N*, we adopt the widely used approach of deriving a diffusion limit for the Markov chain produced by the MALA algorithm. We study this problem for a class of target measures which is *not* in product form and we address the situation of practical relevance in which the algorithm is started out of stationarity. We thereby significantly extend previous works which consider either measures of product form, when the Markov chain is started out of stationarity, or non-product measures (defined via a density with respect to a Gaussian), when the Markov chain is started in stationarity. In order to work in this non-stationary and non-product setting, significant new analysis is required. In particular, our diffusion limit comprises a stochastic PDE coupled to a scalar ordinary differential equation which gives a measure of how far from stationarity the process is. The family of non-product target measures that we consider in this paper are found from discretization of a measure on an infinite dimensional Hilbert space; the discretised measure is defined by its density with respect to a Gaussian random field. The results of this paper demonstrate that, in the non-stationary regime, the cost of the algorithm is of ${{\mathcal {O}}}(N^{1/2})$ in contrast to the stationary regime, where it is of ${{\mathcal {O}}}(N^{1/3})$.

## Introduction

### Context

Metropolis–Hastings algorithms are Markov Chain Monte Carlo (MCMC) methods used to sample from a given probability measure, referred to as the target measure. The basic mechanism consists of employing a proposal transition density *q*(*x*, *y*) in order to produce a reversible Markov chain $\{x^k\}_{k=0}^{\infty }$ for which the target measure $\pi$ is invariant [11]. At step *k* of the chain, a proposal move $y^{k}$ is generated by using *q*(*x*, *y*), i.e. $y^{k} \sim q(x^k, \cdot )$. Then such a move is accepted with probability $\alpha (x^k, y^k)$:

1.1 $$\begin{aligned} \alpha \big (x^k,y^k\big )= \min \left\{ 1, \frac{\pi \big (y^k\big ) q\big (y^k,x^k\big )}{\pi \big (x^k\big ) q\big (x^k,y^k\big )} \right\} . \end{aligned}$$

The computational cost of this algorithm when the state space has high dimension *N* is of practical interest in many applications. The measure of computational cost considered in this paper is the *expected squared jumping distance*, introduced in [19] and related works. Roughly speaking [we will be more precise about this in the next Sect. 1.2, see comments before (1.8)], if the size of the proposal moves is too large, i.e. if we propose moves which are too far away from the current position, then such moves tend to be frequently rejected; on the other hand, if the algorithm proposes moves which are too close to the current position, then such moves will be most likely accepted, however the chain will have not moved very far away. In either extreme cases, the chain tends to get stuck and will exhibit slow mixing, and this is more and more true as the dimension *N* of the state space increases. It is therefore clear that one needs to strike a balance between these two opposite scenarios; in particular, the optimal size of the proposed moves (i.e., the proposal variance) will depend on *N*. If the proposal variance scales with *N* like $N^{-\zeta }$, for some $\zeta >0$, then we will say that the cost of the algorithm, in terms of ESJD, is of the order $N^{\zeta }$.

A widely used approach to tackle this problem is to study diffusion limits for the algorithm. Indeed the scaling used to obtain a well defined diffusion limit corresponds to the optimal scaling of the proposal variance (see Remark 1.1). This problem was first studied in [19], for the Random Walk Metropolis algorithm (RWM); in this work it is assumed that the algorithm is started in stationarity and that the target measure is in product form. In the case of the MALA algorithm, the same problem was considered in [20, 21], again in the stationary regime and for product measures. In this setting, the cost of RWM has been shown to be ${{\mathcal {O}}}(N)$, while the cost of MALA is ${{\mathcal {O}}}(N^{\frac{1}{3}}).$ The same ${{\mathcal {O}}}(N^{\frac{1}{3}})$ scaling for MALA, in the stationary regime, was later obtained in the setting of non-product measures defined via density with respect to a Gaussian random field [17]. In the paper [6] extensions of these results to non-stationary initializations were considered, however only for the Gaussian targets. For Gaussian targets, RWM was shown to scale the same in and out of stationarity, whilst MALA scales like ${{\mathcal {O}}}(N^{\frac{1}{2}})$ out of stationarity. In [12, 13] the RWM and MALA algorithms were studied out of stationarity for quite general product measures and the RWM method shown again to scale the same in and out of stationarity. For MALA the appropriate scaling was shown to differ in and out of stationarity and, crucially, the scaling out of stationarity was shown to depend on a certain moment of the potential defining the product measure. In this paper we contribute further understanding of the MALA algorithm when initialized out of stationarity by considering non-product measures defined via density with respect to a Gaussian random field. Considering such a class of measures has proved fruitful, see e.g. [15, 17]. Relevant to this strand of literature, is also the work [5].

In this paper our primary contribution is the study of diffusion limits for the the MALA algorithm, out of stationarity, in the setting of general non-product measures, defined via density with respect to a Gaussian random field. Significant new analysis is needed for this problem because the work of [17] relies heavily on stationarity in analyzing the acceptance probability, whilst the work of [13] uses propagation of chaos techniques, unsuitable for non-product settings.

The challenging diffusion limit obtained in this paper is relevant both to the picture just described and, in general, due to the widespread practical use of the MALA algorithm. The understanding we obtain about the MALA algorithm when applied to realistic non-product targets is one of the main motivations for the analysis that we undertake in this paper. The diffusion limit we find is given by an SPDE coupled to a one-dimensional ODE. The evolution of such an ODE can be taken as an indicator of how close the chain is to stationarity (see Remark 1.1 for more details on this). The scaling adopted to obtain such a diffusion limit shows that the cost of the algorithm is of order $N^{1/2}$ in the non-stationary regime, as opposed to what happens in the stationary phase, where the cost is of order $N^{1/3}$. It is important to recognize that, for measures absolutely continuous with respect to a Gaussian random field, algorithms exist which require ${{\mathcal {O}}}(1)$ steps in and out of stationarity; see [7] for a review. Such methods were suggested by Radford Neal in [16], and developed by Alex Beskos for conditioned stochastic differential equations in [4], building on the general formulation of Metropolis–Hastings methods in [23]; these methods are analyzed from the point of view of diffusion limits in [18]. It thus remains open and interesting to study the MALA algorithm out of stationarity for non-product measures which are not defined via density with respect to a Gaussian random field; however the results in [12] demonstrate the substantial technical barriers that will exist in trying to do so. An interesting starting point of such work might be the study of non i.i.d. product measures as pioneered by Bédard [2, 3].

### Setting and the main result

Let (${\mathcal {H}}, \langle \cdot , \cdot \rangle , \Vert \cdot \Vert$) be an infinite dimensional separable Hilbert space and consider the measure $\pi$ on ${\mathcal {H}}$, defined as follows:

1.2 $$\begin{aligned} \frac{d\pi }{d\pi _0} \propto \exp ({-\varPsi }), \qquad \pi _0:={\mathcal {N}}(0,{\mathcal {C}}). \end{aligned}$$

That is, $\pi$ is absolutely continuous with respect to a Gaussian measure $\pi _0$ with mean zero and covariance operator ${\mathcal {C}}$. $\varPsi$ is some real valued functional with domain ${\tilde{{\mathcal {H}}}} \subseteq {\mathcal {H}}$, $\varPsi : {\tilde{{\mathcal {H}}}}\rightarrow {\mathbb {R}}$. Measures of the form (1.2) naturally arise in Bayesian nonparametric statistics and in the study of conditioned diffusions [10, 22]. In Sect. 2 we will give the precise definition of the space ${\tilde{{\mathcal {H}}}}$ and identify it with an appropriate Sobolev-like subspace of ${\mathcal {H}}$ (denoted by ${\mathcal {H}}^s$ in Sect. 2).The covariance operator ${\mathcal {C}}$ is a positive, self-adjoint, trace class operator on ${\mathcal {H}}$, with eigenbasis $\{\lambda _j^2, \phi _j\}$:

1.3 $$\begin{aligned} {\mathcal {C}}\phi _j= \lambda _j^2 \phi _j, \quad \forall j \in {\mathbb {N}}, \end{aligned}$$

and we assume that the set $\{\phi _j\}_{j \in {\mathbb {N}}}$ is an orthonormal basis for ${\mathcal {H}}$.

We will analyse the MALA algorithm designed to sample from the finite dimensional projections $\pi ^N$ of the measure (1.2) on the space

1.4 $$\begin{aligned} X^N:=\text {span}\{\phi _j\}_{j=1}^N \subset {\mathcal {H}}\end{aligned}$$

spanned by the first *N* eigenvectors of the covariance operator. Notice that the space $X^N$ is isomorphic to ${\mathbb {R}}^N$. To clarify this further, we need to introduce some notation. Given a point $x \in {\mathcal {H}}$, ${\mathcal {P}}^N(x):=\sum _{j=1}^n\left\langle \phi _j,x \right\rangle \phi _j$ is the projection of *x* onto the space $X^N$ and we define the approximations of functional $\varPsi$ and covariance operator ${\mathcal {C}}$:

1.5 $$\begin{aligned} \varPsi ^N:=\varPsi \circ {\mathcal {P}}^N \quad \text{ and } \quad {\mathcal {C}}_N:={\mathcal {P}}^N\circ {\mathcal {C}}\circ {\mathcal {P}}^N. \end{aligned}$$

With this notation in place, our target measure is the measure $\pi ^N$ (on $X^N \cong {\mathbb {R}}^N$) defined as

1.6 $$\begin{aligned} \frac{d\pi ^N}{d\pi _0^N}(x)=M_{\varPsi ^N}e^{-\varPsi ^N(x)}, \qquad \pi _0^N:={\mathcal {N}}(0,{\mathcal {C}}_N), \end{aligned}$$

where $M_{\varPsi ^N}$ is a normalization constant. Notice that the sequence of measures $\{\pi ^N\}_{N\in {\mathbb {N}}}$ approximates the measure $\pi$ (in particular, the sequence $\{\pi ^N\}_{N\in {\mathbb {N}}}$ converges to $\pi$ in the Hellinger metric, see [22, Section 4] and references therein). In order to sample from the measure $\pi ^N$ in (1.6), we will consider the MALA algorithm with proposal

1.7 $$\begin{aligned} y^{k,N}=x^{k,N}+\delta {\mathcal {C}}_N\nabla \log \pi ^N\big (x^{k,N}\big )+ \sqrt{2 \delta }\, {\mathcal {C}}_N^{1/2} \xi ^{k,N}, \end{aligned}$$

where

$$\begin{aligned} \xi ^{k,N}=\sum _{i=1}^N \xi _i\phi _i, \quad \xi _i {\mathop {\sim }\limits ^{{\mathcal {D}}}} {\mathcal {N}}(0,1) \text{ i.i.d. }, \end{aligned}$$

and $\delta >0$ is a positive parameter. We rewrite $y^{k,N}$ as

$$\begin{aligned} y^{k,N}=x^{k,N}-\delta \bigl (x^{k,N}+ {\mathcal {C}}_N \nabla \varPsi ^N\big (x^{k,N}\big )\bigr )+ \sqrt{2 \delta }\, {\mathcal {C}}_N^{1/2} \xi ^{k,N}. \end{aligned}$$

The proposal defines the kernel *q* and enters in the accept-reject criterion $\alpha$, which is added to preserve detailed balance with respect to $\pi ^N$ (more details on the algorithm will be given in Sect. 2.2). The proposal is a discretization of a $\pi ^N$-invariant diffusion process with time step $\delta$; in the MCMC literature $\delta$ is often referred to as the proposal variance. The accept-reject criterion compensates for the discretization, which destroys the $\pi ^N$-reversibility. A crucial parameter to be appropriately chosen in order to optimize the performance of the algorithm is $\delta$; such a choice will depend on the dimension *N* of the state space. To be more precise, set $\delta =\ell N^{-\zeta }$, where $\ell , \zeta$ are two positive parameters, the latter being, for the time, the most relevant to this discussion. As explained when outlining the context of this paper, if $\zeta$ is too large (so that $\delta$ is too small) then the algorithm will tend to move very slowly; if $\zeta$ is too big, then the proposed moves will be very large and the algorithm will tend to reject them very often. In this paper we show that, if the algorithm is started our of stationarity then, in the non-stationary regime, the optimal choice of $\zeta$ is $\zeta =1/2$. In particular, if

1.8 $$\begin{aligned} \delta =\ell /\sqrt{N} \end{aligned}$$

then the acceptance probability is ${{\mathcal {O}}}(1)$. Furthermore, starting from the Metropolis–Hastings chain $\{x^{k,N}\}_{k\in {\mathbb {N}}}$, we define the continuous interpolant

1.9 $$\begin{aligned}&x^{(N)}(t)=(N^{1/2}t-k)x^{k+1,N}+(k+1-N^{1/2}t)x^{k,N}, \quad \nonumber \\&t_k\le t< t_{k+1}, \text{ where } t_k=\frac{k}{N^{1/2}}. \end{aligned}$$

This process converges weakly to a diffusion process. The precise statement of such a result is given in Theorem 4.2 (and Sect. 4 contains heuristic arguments which explain how such a result is obtained). In proving the result we will use the fact that *W*(*t*) is a ${\mathcal {H}}_s$-valued Brownian motion with covariance ${\mathcal {C}}_s$, with ${\mathcal {H}}_s$ a (Hilbert) subspace of ${\mathcal {H}}$ and ${\mathcal {C}}_s$ the covariance in this space. Details of these spaces are given in Sect. 2, see in particular (2.4) and (2.5). Below $C([0,T];{\mathcal {H}}_s)$ denotes the space of ${\mathcal {H}}_s$-valued continuous functions on [0, *T*], endowed with the uniform topology; $\alpha _{\ell }, h_{\ell }$ and $b_{\ell }$ are real valued functions, which we will define immediately after the statement, and $x^{k,N}_j$ denotes the *j*th component of the vector $x^{k,N}\in X^N$ with respect to the basis $\{\phi _1,\ldots ,\phi _N\}$ (more details on this notation are given in Sect. 2.1.)

#### Main Result

Let $\{x^{k,N}\}_{k\in {\mathbb {N}}}$ be the Metropolis–Hastings Markov chain to sample from $\pi ^N$ and constructed using the MALA proposal (1.7) (i.e. the chain (2.14)) with $\delta$ chosen to satisfy (1.8). Then, for any deterministic initial datum $x^{0,N}={\mathcal {P}}^N(x^0)$, where $x^0$ is any point in ${\mathcal {H}}_s$, the continuous interpolant $x^{(N)}$ defined in (1.9) converges weakly in $C([0,T];{\mathcal {H}}_s)$ to the solution of the SDE

1.10 $$\begin{aligned} dx(t)=- h_{\ell }(S(t)) \bigl (x(t)+{\mathcal {C}}\nabla \varPsi (x(t)) \bigr ) \, dt+\sqrt{2h_{\ell }(S(t))} \, dW(t) , \quad x(0)=x^0, \end{aligned}$$

where $S(t) \in {\mathbb {R}}_+:=\{s\in {\mathbb {R}}: s\ge 0\}$ solves the ODE

1.11 $$\begin{aligned} dS(t)=b_{\ell }(S(t))\, dt, \qquad S(0):= \lim _{N \rightarrow \infty } \frac{1}{N}\sum _{j=1}^N \frac{\left| x_j^{0,N} \right| ^2}{\lambda _j^2} . \end{aligned}$$

In the above the initial datum *S*(0) is assumed to be finite and *W*(*t*) is a ${\mathcal {H}}_s$-valued Brownian motion with covariance ${\mathcal {C}}_s$.

The functions $\alpha _{\ell }, h_{\ell }, b_{\ell }: {\mathbb {R}}\rightarrow {\mathbb {R}}$ in the previous statement are defined as follows:

1.12 $$\begin{aligned} \alpha _{\ell }(s)&= 1\wedge e^{\ell ^2 (s-1)/2} \end{aligned}$$

1.13 $$\begin{aligned} h_{\ell }(s)&= \ell \alpha _{\ell }(s) \end{aligned}$$

1.14 $$\begin{aligned} b_{\ell }(s)&= 2\ell (1-s)\left( 1\wedge e^{\ell ^2 (s-1)/2} \right) = 2 (1-s) h_{\ell }(s). \end{aligned}$$

#### Remark 1.1

We make several remarks concerning the main result.

- Since the effective time-step implied by the interpolation (1.9) is $N^{-1/2}$, the main result implies that the number of steps required by the Markov chain in its non-stationary regime is ${{\mathcal {O}}}(N^{1/2})$. A more detailed discussion on this fact can be found in Sect. 4.
- Notice that Eq. (1.11) evolves independently of Eq. (1.10). Once the MALA algorithm (2.14) is introduced and an initial state $x^0\in {\tilde{{\mathcal {H}}}}$ is given such that *S*(0) is finite, the real valued (double) sequence $S^{k,N}$,
  1.15 $$\begin{aligned} S^{k,N}:=\frac{1}{N} \sum _{i=1}^N \frac{\left| x^{k,N}_i\right| ^2}{\lambda _i^2} \end{aligned}$$
  started at $S_0^N:=\frac{1}{N} \sum _{i=1}^N \frac{\left| x^{0,N}_i\right| ^2}{\lambda _i^2}$ is well defined. For fixed *N*, $\{S^{k,N}\}_k$ is not, in general, a Markov process (however it is Markov if e.g. $\varPsi =0$). Consider the continuous interpolant $S^{(N)}(t)$ of the sequence $S^{k,N}$, namely
  1.16 $$\begin{aligned} S^{(N)}(t)=(N^{1/2}t-k)S^{k+1,N}+(k+1-N^{1/2}t)S^{k,N}, \quad t_k\le t< t_{k+1}, \,\, t_k=\frac{k}{N^{\frac{1}{2}}}.\nonumber \\ \end{aligned}$$
  In Theorem 4.1 we prove that $S^{(N)}(t)$ converges in probability in $C([0,T];{\mathbb {R}})$ to the solution of the ODE (1.11) with initial condition $S_0:=\lim _{N\rightarrow \infty }S_0^N$. Once such a result is obtained, we can prove that $x^{(N)}(t)$ converges to *x*(*t*). We want to stress that the convergence of $S^{(N)}(t)$ to *S*(*t*) can be obtained independently of the convergence of $x^{(N)}(t)$ to *x*(*t*).
- Let $S(t):{\mathbb {R}}\rightarrow {\mathbb {R}}$ be the solution of the ODE (1.11). We will prove (see Theorem 3.1) that $S(t) \rightarrow 1$ as $t\rightarrow \infty$; this is also consistent with the fact that, in stationarity, $S^{k,N}$ converges to 1 as $N \rightarrow \infty$ (for every $k>0$), see Remark 4.1. In view of this and the above comment, *S*(*t*) (or $S^{k,N}$) can be taken as an indication of how close the chain is to stationarity. Moreover, notice that $h_{\ell }(1)=\ell$; heuristically one can then argue that the asymptotic behaviour of the law of *x*(*t*), the solution of (1.10), is described by the law of the following infinite dimensional SDE:
  1.17 $$\begin{aligned} dz(t)=-\ell (z(t)+{\mathcal {C}}\nabla \varPsi (z(t)))dt+ \sqrt{2\ell } dW(t). \end{aligned}$$
  It was proved in [9, 10] that (1.17) is ergodic with unique invariant measure given by (1.2). Our deduction concerning computational cost is made on the assumption that the law of (1.10) does indeed tend to the law of (1.17), although we will not prove this here as it would take us away from the main goal of the paper which is to establish the diffusion limit of the MALA algorithm.
- In [12, 13] the diffusion limit for the MALA algorithm started out of stationarity and applied to i.i.d. target product measures is given by a non-linear equation of McKean-Vlasov type. This is in contrast with our diffusion limit, which is an infinite-dimensional SDE. The reason why this is the case is discussed in detail in [14, Section 1.2]. The discussion in the latter paper is in the context of the Random Walk Metropolis algorithm, but it is conceptually analogous to what holds for the MALA algorithm and for this reason we do not spell it out here.
- In this paper we make stronger assumptions on $\varPsi$ than are required to prove a diffusion limit in the stationary regime [17]. In particular we assume that the first derivative of $\varPsi$ is bounded, whereas [17] requires only boundedness of the second derivative. Removing this assumption on the first derivative, or showing that it is necessary, would be of interest but would require different techniques to those employed in this paper and we do not address the issue here.

#### Remark 1.2

The proposal we employ in this paper is the standard MALA proposal. It can be seen as a particular case of the more general proposal introduced in [4, equation (4.2)] see also [1]; in our notation this proposal can be written as

1.18 $$\begin{aligned} y^{k+1,N}= x^{k,N} +\delta \big \{-(1-\theta )x^{k,N}-\theta y^{k+1,N}- {\mathcal {C}}_N \nabla \varPsi ^N \big (x^{k,N}\big )\big \}+\sqrt{2 \delta } \xi ^{k,N}.\nonumber \\ \end{aligned}$$

In the above, $\theta \in [0,1]$ is a parameter. The choice $\theta = 0$ corresponds to our proposal. When $\theta = 1/2$, the resulting algorithm is well posed in infinite dimensions; as a consequence a diffusion limit is obtained, in and out of stationarity, without scaling $\delta$ with respect to *N*; see Remark 4.3. When $\theta \ne 1/2$ the algorithms all suffer from the curse of dimensionality: it is necessary to scale $\delta$ inversely with a power of *N* to obtain an acceptable acceptance probability. In this paper we study how the efficiency decreases with *N* when $\theta =0$; results analogous to the one we prove here will hold for any $\theta \ne 1/2$, but proving them at this level of generality would lengthen the article without adding insight. Furthermore, for non-Gaussian priors practitioners might use the algorithm with $\theta =0$ and so our results shed light on that case; if the prior is actually Gaussian practitioners should use the algorith with $\theta = \frac{1}{2}.$ There is no reasons to use any other values of $\theta$ in practice, as far as we are aware.

### Structure of the paper

The paper is organized as follows. In Sect. 2 we introduce the notation and the assumptions that we use throughout this paper. In particular, Sect. 2.1 introduces the infinite dimensional setting in which we work, Sect. 2.2 discusses the MALA algorithm and the assumptions we make on the functional $\varPsi$ and on the covariance operator ${\mathcal {C}}$. Section 3 contains the proof of existence and uniqueness of solutions for the limiting Eqs. (1.10) and (1.11). With these preliminaries in place, we give, in Sect. 4, the formal statement of the main results of this paper, Theorems 4.1 and 4.2. In this section we also provide heuristic arguments outlining how the main results are obtained. The complete proof of these results builds on a continuous mapping argument presented in Sect. 5. The heuristics of Sect. 4 are made rigorous in Sects. 6–8. In particular, Sect. 6 contains some estimates of the size of the chain’s jumps and the growth of its moments, as well as the study of the acceptance probability. In Sects. 7 and 8 we use these estimates and approximations to prove Theorems 4.1 and 4.2, respectively. Readers interested in the structure of the proofs of Theorems 4.1 and 4.2 but not in the technical details may wish to skip the ensuing two sections (Sects. 2 and 3) and proceed directly to the statement of these results and the relevant heuristics discussed in Sect. 4.

## Notation, algorithm, and assumptions

In this section we detail the notation and the assumptions (Sects. 2.1 and 2.3, respectively) that we will use in the rest of the paper.

### Notation

Let $\left( {\mathcal {H}}, \langle \cdot , \cdot \rangle , \Vert \cdot \Vert \right)$ denote a real separable infinite dimensional Hilbert space, with the canonical norm induced by the inner-product. Let $\pi _0$ be a zero-mean Gaussian measure on ${\mathcal {H}}$ with covariance operator ${\mathcal {C}}$. By the general theory of Gaussian measures [8], ${\mathcal {C}}$ is a positive, trace class operator. Let $\{\phi _j,\lambda ^2_j\}_{j \ge 1}$ be the eigenfunctions and eigenvalues of ${\mathcal {C}}$, respectively, so that (1.3) holds. We assume a normalization under which $\{\phi _j\}_{j \ge 1}$ forms a complete orthonormal basis of ${\mathcal {H}}$. Recalling (1.4), we specify the notation that will be used throughout this paper:

- *x* and *y* are elements of the Hilbert space ${\mathcal {H}}$;
- the letter *N* is reserved to denote the dimensionality of the space $X^N$ where the target measure $\pi ^N$ is supported;
- $$x^N$$ is an element of $$X^N$$$$\cong {\mathbb {R}}^N$$ (similarly for $$y^N$$ and the noise $$\xi ^N$$);
- for any fixed $N \in {\mathbb {N}}$, $x^{k,N}$ is the *k*th step of the chain $\{x^{k,N}\}_{k \in {\mathbb {N}}} \subseteq X^N$ constructed to sample from $\pi ^N$; $x^{k,N}_i$ is the *i*th component of the vector $x^{k,N}$, that is $x^{k,N}_i:=\langle x^{k,N}, \phi _i\rangle$ (with abuse of notation).

For every $x \in {\mathcal {H}}$, we have the representation $x = \sum _{j\ge 1} \; x_j \phi _j$, where $x_j:=\langle x,\phi _j\rangle .$ Using this expansion, we define Sobolev-like spaces ${\mathcal {H}}^s, s \in {\mathbb {R}}$, with the inner-products and norms defined by

$$\begin{aligned} \langle x,y \rangle _s = \sum _{j=1}^\infty j^{2s}x_jy_j \qquad \text {and} \qquad \Vert x\Vert ^2_s = \sum _{j=1}^\infty j^{2s} \, x_j^{2}. \end{aligned}$$

The space $({\mathcal {H}}^s, \langle \cdot , \cdot \rangle _s)$ is also a Hilbert space. Notice that ${\mathcal {H}}^0 = {\mathcal {H}}$. Furthermore ${\mathcal {H}}^s \subset {\mathcal {H}}\subset {\mathcal {H}}^{-s}$ for any $s >0$. The Hilbert–Schmidt norm $\Vert \cdot \Vert _{\mathcal {C}}$ associated with the covariance operator ${\mathcal {C}}$ is defined as

$$\begin{aligned} \left| \left| x\right| \right| _{{\mathcal {C}}}^2 := \sum _{j=1}^{\infty } \lambda _j^{-2} x_j^2= \sum _{j=1}^{\infty } \frac{\left| \langle x, \phi _j\rangle \right| ^2}{\lambda _j^2},\qquad x\in {\mathcal {H}}, \end{aligned}$$

and it is the Cameron–Martin norm associated with the Gaussian measure ${\mathcal {N}}(0,{\mathcal {C}})$. Such a norm is induced by the scalar product

$$\begin{aligned} \langle x, y\rangle _{{\mathcal {C}}} :=\langle {\mathcal {C}}^{-1/2}x, {\mathcal {C}}^{-1/2}y \rangle , \qquad x,y\in {\mathcal {H}}. \end{aligned}$$

Similarly, ${\mathcal {C}}_N$ defines a Hilbert–Schmidt norm on $X^N$,

2.1 $$\begin{aligned} \left| \left| x^N\right| \right| _{{\mathcal {C}}_N}^2:=\sum _{j=1}^{N} \frac{\left| \langle x^N, \phi _j\rangle \right| ^2}{\lambda _j^2},\qquad x^N\in X^N, \end{aligned}$$

which is induced by the scalar product

$$\begin{aligned} \langle x^N, y^N\rangle _{{\mathcal {C}}_N} :=\left\langle {\mathcal {C}}_N^{-1/2}x^N, {\mathcal {C}}_N^{-1/2}y^N \right\rangle , \qquad x^N,y^N\in X^N. \end{aligned}$$

For $s \in {\mathbb {R}}$, let $L_s : {\mathcal {H}}\rightarrow {\mathcal {H}}$ denote the operator which is diagonal in the basis $\{\phi _j\}_{j \ge 1}$ with diagonal entries $j^{2s}$,

$$\begin{aligned} L_s \,\phi _j = j^{2s} \phi _j, \end{aligned}$$

so that $L^{\frac{1}{2}}_s \,\phi _j = j^s \phi _j$. The operator $L_s$ lets us alternate between the Hilbert space ${\mathcal {H}}$ and the interpolation spaces ${\mathcal {H}}^s$ via the identities:

$$\begin{aligned} \langle x,y \rangle _s = \left\langle L^{\frac{1}{2}}_s x,L^{\frac{1}{2}}_s y \right\rangle \qquad \text {and} \qquad \Vert x\Vert ^2_s =\left\| L^{\frac{1}{2}}_s x\right\| ^2. \end{aligned}$$

Since $\left| \left| L_s^{-1/2} \phi _k\right| \right| _{s} = \left| \left| \phi _k\right| \right| =1$, we deduce that $\{{\hat{\phi }}_k:=L^{-1/2}_s \phi _k \}_{k \ge 1}$ forms an orthonormal basis of ${\mathcal {H}}^s$. An element $y\sim {\mathcal {N}}(0,{\mathcal {C}})$ can be expressed as

2.2 $$\begin{aligned} y=\sum _{j=1}^{\infty } \lambda _j \rho _j \phi _j \qquad \text{ with } \qquad \rho _j{\mathop {\sim }\limits ^{{\mathcal {D}}}}{\mathcal {N}}(0,1) \,\,\text{ i.i.d }. \end{aligned}$$

If $\sum _j \lambda _j^2 j^{2s}<\infty$, then *y* can be equivalently written as

2.3 $$\begin{aligned} y=\sum _{j=1}^{\infty } (\lambda _j j^s) \rho _j (L_s^{-1/2} \phi _j) \qquad \text{ with } \qquad \rho _j{\mathop {\sim }\limits ^{{\mathcal {D}}}}{\mathcal {N}}(0,1) \,\,\text{ i.i.d }. \end{aligned}$$

For a positive, self-adjoint operator $D : {\mathcal {H}}\mapsto {\mathcal {H}}$, its trace in ${\mathcal {H}}$ is defined as

$$\begin{aligned} {\mathrm{Trace}}_{{\mathcal {H}}}(D) \;{:=}\; \sum _{j=1}^\infty \langle \phi _j, D \phi _j \rangle . \end{aligned}$$

We stress that in the above $\{ \phi _j \}_{j \in {\mathbb {N}}}$ is an orthonormal basis for $({\mathcal {H}}, \langle \cdot , \cdot \rangle )$. Therefore, if ${\tilde{D}}:{\mathcal {H}}^s \rightarrow {\mathcal {H}}^s$, its trace in ${\mathcal {H}}^s$ is

$$\begin{aligned} {\mathrm{Trace}}_{{\mathcal {H}}^s}({\tilde{D}}) \;{=}\; \sum _{j=1}^\infty \left\langle L_s^{-\frac{1}{2}} \phi _j, {\tilde{D}} L_s^{-\frac{1}{2}} \phi _j \right\rangle _s. \end{aligned}$$

Since ${\mathrm{Trace}}_{{\mathcal {H}}^s}({\tilde{D}})$ does not depend on the orthonormal basis, the operator ${\tilde{D}}$ is said to be trace class in ${\mathcal {H}}^s$ if ${\mathrm{Trace}}_{{\mathcal {H}}^s}({\tilde{D}}) < \infty$ for some, and hence any, orthonormal basis of ${\mathcal {H}}^s$. Because ${\mathcal {C}}$ is defined on ${\mathcal {H}}$, the covariance operator^1^

2.4 $$\begin{aligned} {\mathcal {C}}_s=L_s^{1/2} {\mathcal {C}}L_s^{1/2} \end{aligned}$$

is defined on ${\mathcal {H}}^s$. Thus, for all the values of *r* such that ${\mathrm{Trace}}_{{\mathcal {H}}^s}({\mathcal {C}}_s)=\sum _j \lambda _j^2 j^{2s}< \infty$, we can think of *y* as a mean zero Gaussian random variable with covariance operator ${\mathcal {C}}$ in ${\mathcal {H}}$ and ${\mathcal {C}}_s$ in ${\mathcal {H}}^s$ [see (2.2) and (2.3)]. In the same way, if ${\mathrm{Trace}}_{{\mathcal {H}}^s}({\mathcal {C}}_s)< \infty$, then

2.5 $$\begin{aligned} W(t)= \sum _{j=1}^{\infty } \lambda _j w_j(t) \phi _j= \sum _{j=1}^{\infty }\lambda _j j^r w_j(t) {\hat{\phi }}_j, \end{aligned}$$

where $\{ w_j(t)\}_{j \ge 1}$ a collection of i.i.d. standard Brownian motions on ${\mathbb {R}}$, can be equivalently understood as an ${\mathcal {H}}$-valued ${\mathcal {C}}$-Brownian motion or as an ${\mathcal {H}}^s$-valued ${\mathcal {C}}_s$-Brownian motion.

We will make use of the following elementary inequality,

2.6 $$\begin{aligned} \left| \left\langle x,y \right\rangle \right| ^2=\left| \sum _{j=1}^{\infty } (j^s x_j)(j^{-s}y_j)\right| ^2 \le \left| \left| x\right| \right| _{s}^2 \left| \left| y\right| \right| _{-s}^2,\qquad \forall x \in {\mathcal {H}}^s,\quad y \in {\mathcal {H}}^{-s}.\nonumber \\ \end{aligned}$$

Throughout this paper we study sequences of real numbers, random variables and functions, indexed by either (or both) the dimension *N* of the space on which the target measure is defined or the chain’s step number *k*. In doing so, we find the following notation convenient.

- Two (double) sequences of real numbers $\{A^{k,N}\}$ and $\{B^{k,N}\}$ satisfy $A^{k,N} \lesssim B^{k,N}$ if there exists a constant $K>0$ (independent of *N* and *k*) such that
  $$\begin{aligned} A^{k,N}\le KB^{k,N}, \end{aligned}$$
  for all *N* and *k* such that $\{A^{k,N}\}$ and $\{B^{k,N}\}$ are defined.
- If the $A^{k,N}$s and $B^{k,N}$s are random variables, the above inequality must hold almost surely (for some deterministic constant *K*).
- If the $A^{k,N}$s and $B^{k,N}$s are real-valued functions on ${\mathcal {H}}$ or ${\mathcal {H}}^s$, $A^{k,N}= A^{k,N}(x)$ and $B^{k,N}= B^{k,N}(x)$, the same inequality must hold with *K* independent of *x*, for all *x* where the $A^{k,N}$s and $B^{k,N}$s are defined.

As is customary, ${\mathbb {R}}_+:=\{s\in {\mathbb {R}}: s \ge 0\}$ and for all $b \in {\mathbb {R}}_+$ we let $[b]=n$ if $n\le b < n+1$ for some integer *n*. Finally, for time dependent functions we will use both the notations *S*(*t*) and $S_t$ interchangeably.

### The algorithm

A natural variant of the MALA algorithm stems from the observation that $\pi ^N$ is the unique stationary measure of the SDE

2.7 $$\begin{aligned} dY_t={\mathcal {C}}_N\nabla \log \pi ^N(Y_t)dt+\sqrt{2}dW^N_t, \end{aligned}$$

where $W^N$ is an $X^N$-valued Brownian motion with covariance operator ${\mathcal {C}}_N$. The algorithm consists of discretising (2.7) using the Euler-Maruyama scheme and adding a Metropolis accept-reject step so that the invariance of $\pi ^N$ is preserved. The variant on MALA which we study is therefore a Metropolis–Hastings algorithm with proposal

2.8 $$\begin{aligned} y^{k,N} =x^{k,N}- \delta \left( x^{k,N}+ {\mathcal {C}}_N \nabla \varPsi ^N\big (x^{k,N}\big )\right) + \sqrt{2\delta } {\mathcal {C}}_N^{1/2} \xi ^{k,N}, \end{aligned}$$

where

$$\begin{aligned} \xi ^{k,N}:= \sum _{j=1}^N \xi ^{k,N}_j \phi _j, \quad \xi ^{k,N}_j \sim {\mathcal {N}}(0,1){ \text{ i.i.d }}. \end{aligned}$$

We stress that the Gaussian random variables $\xi ^{k,N}_i$ are independent of each other and of the current position $x^{k,N}$. Motivated by the considerations made in the introduction (and that will be made more explicit in Sect. 4.1), in this paper we fix the choice

2.9 $$\begin{aligned} \delta :=\frac{\ell }{N^{1/2}}. \end{aligned}$$

If at step *k* the chain is at $x^{k,N}$, the algorithm proposes a move to $y^{k,N}$ defined by Eq. (2.8). The move is then accepted with probability

2.10 $$\begin{aligned} \alpha ^N\big (x^{k,N},y^{k,N}\big ):=\frac{\pi ^N\big (y^{k,N}\big ) q^N\big (y^{k,N}, x^{k,N}\big )}{\pi ^N\big (x^{k,N}\big ) q^N\big (x^{k,N}, y^{k,N}\big )}, \end{aligned}$$

where, for any $x^N, y^N \in {\mathbb {R}}^N \simeq X^N$,

2.11 $$\begin{aligned} q^N\big (x^N,y^N\big )\propto e^{-\frac{1}{4\delta }\Vert \big (y^N-x^N\big )-\delta \nabla \log \pi ^N\big (x^N\big )\Vert ^2_{{\mathcal {C}}_N}}. \end{aligned}$$

If the move to $y^{k,N}$ is accepted then $x^{k+1,N}=y^{k,N}$, if it is rejected the chain remains where it was, i.e. $x^{k+1,N}=x^{k,N}$. In short, the MALA chain is defined as follows:

2.12 $$\begin{aligned} x^{k+1,N}:=\gamma ^{k,N} y^{k,N}+ \big (1-\gamma ^{k,N})x^{k,N},\qquad x^{0,N}:={\mathcal {P}}^N(x^0\big ), \end{aligned}$$

where in the above

2.13 $$\begin{aligned} \gamma ^{k,N}{\mathop {\sim }\limits ^{{\mathcal {D}}}}{\mathrm{Bernoulli}}\big ( \alpha ^N\big (x^{k,N},y^{k,N}\big )\big ); \end{aligned}$$

that is, conditioned on $(x^{k,N},y^{k,N})$, $\gamma ^{k,N}$ has Bernoulli law with mean $\alpha ^N(x^{k,N},y^{k,N})$. Equivalently, we can write

$$\begin{aligned} \gamma ^{k,N}=\mathbf{{1}}_{\big \{U^{k,N}\le \alpha ^N\big (x^{k,N},y^{k,N}\big )\big \}}, \end{aligned}$$

with $U^{k,N}{\mathop {\sim }\limits ^{{\mathcal {D}}}}$ Uniform$\,[0,1]$, independent of $x^{k,N}$ and $\xi ^{k,N}$.

For fixed *N*, the chain $\{x^{k,N}\}_{k\ge 1}$ lives in $X^N \cong {\mathbb {R}}^N$ and samples from $\pi ^N$. However, in view of the fact that we want to study the scaling limit of such a chain as $N \rightarrow \infty$, the analysis is cleaner if it is carried out in ${\mathcal {H}}$; therefore, the chain that we analyse is the chain $\{x^k\}_{k}\subseteq {\mathcal {H}}$ defined as follows: the first *N* components of the vector $x^k \in {\mathcal {H}}$ coincide with $x^{k,N}$ as defined above; the remaining components are not updated and remain equal to their initial value. More precisely, using (2.8) and (2.12), the chain $x^k$ can be written in a component-wise notation as follows:

2.14 $$\begin{aligned}&x^{k+1}_i=x^{k+1,N}_i =x^{k,N}_i- \gamma ^{k,N} \left[ \frac{\ell }{N^{1/2}}\left( x^{k,N}_i+ \big [{\mathcal {C}}_N \nabla \varPsi ^N\big (x^{k,N}\big )\big ]_i\right) \right. \nonumber \\&\qquad \qquad \qquad \qquad \qquad \left. + \sqrt{\frac{2 \ell }{N^{1/2}}} \lambda _i \,\xi ^{k,N} \right] \qquad \forall i\le N \end{aligned}$$

and

2.15 $$\begin{aligned} x_i^{k+1}&=x^k_i=0 \qquad \forall i\ge N+1. \end{aligned}$$

For the sake of clarity, we specify that $[{\mathcal {C}}_N \nabla \varPsi ^N(x^{k,N})]_i$ denotes the *i*th component of the vector ${\mathcal {C}}_N \nabla \varPsi ^N(x^{k,N}) \in {\mathcal {H}}^s$. From the above it is clear that the update rule (2.14) only updates the first *N* coordinates (with respect to the eigenbasis of ${\mathcal {C}}$) of the vector $x^k$. Therefore the algorithm evolves in the finite-dimensional subspace $X^N$. From now on we will avoid using the notation $\{x^k\}_k$ for the “extended chain” defined in ${\mathcal {H}}$, as it can be confused with the notation $x^N$, which instead is used throughout to denote a generic element of the space $X^N$.

We conclude this section by remarking that, if $x^{k,N}$ is given, the proposal $y^{k,N}$ only depends on the Gaussian noise $\xi ^{k,N}$. Therefore the acceptance probability will be interchangeably denoted by $\alpha ^N\big (x^N,y^N\big )$ or $\alpha ^N\big (x^N,\xi ^N\big )$.

### Assumptions

In this section, we describe the assumptions on the covariance operator ${\mathcal {C}}$ of the Gaussian measure $\pi _0 {\mathop {\sim }\limits ^{{\mathcal {D}}}}{\mathcal {N}}(0,{\mathcal {C}})$ and those on the functional $\varPsi$. We fix a distinguished exponent $s\ge 0$ and assume that $\varPsi : {\mathcal {H}}^s\rightarrow {\mathbb {R}}$ and ${\mathrm{Trace}}_{{\mathcal {H}}^s}({\mathcal {C}}_s)<\infty$. In other words, ${\mathcal {H}}^s$ is the space that we were denoting with ${\tilde{{\mathcal {H}}}}$ in the introduction. Since

2.16 $$\begin{aligned} {\mathrm{Trace}}_{{\mathcal {H}}^s}({\mathcal {C}}_s)= \sum _{j=1}^{\infty } \lambda _j^2 j^{2s}, \end{aligned}$$

the condition ${\mathrm{Trace}}_{{\mathcal {H}}^s}({\mathcal {C}}_s)<\infty$ implies that $\lambda _j j^s \rightarrow 0$ as $j \rightarrow \infty$. Therefore the sequence $\{\lambda _j j^s\}_j$ is bounded:

2.17 $$\begin{aligned} \lambda _j j^s \le C, \end{aligned}$$

for some constant $C>0$ independent of *j*.

For each $x \in {\mathcal {H}}^s$ the derivative $\nabla \varPsi (x)$ is an element of the dual ${\mathcal {L}}({\mathcal {H}}^s,{\mathbb {R}})$ of ${\mathcal {H}}^s$, comprising the linear functionals on ${\mathcal {H}}^s$. However, we may identify ${\mathcal {L}}({\mathcal {H}}^s,{\mathbb {R}})={\mathcal {H}}^{-s}$ and view $\nabla \varPsi (x)$ as an element of ${\mathcal {H}}^{-s}$ for each $x \in {\mathcal {H}}^s$. With this identification, the following identity holds

2.18 $$\begin{aligned} \left| \left| \nabla \varPsi (x)\right| \right| _{{\mathcal {L}}({\mathcal {H}}^s,{\mathbb {R}})} = \left| \left| \nabla \varPsi (x)\right| \right| _{-s}. \end{aligned}$$

To avoid technical complications we assume that the gradient of $\varPsi (x)$ is bounded and globally Lipschitz. More precisely, throughout this paper we make the following assumptions.

#### Assumption 2.1

The functional $\varPsi$ and covariance operator ${\mathcal {C}}$ satisfy the following:

1. **Decay of Eigenvalues** $\lambda _j^2$ of ${\mathcal {C}}$: there exists a constant $\kappa > s+\frac{1}{2}$ such that
  $$\begin{aligned} j^{-\kappa }\lesssim \lambda _j \lesssim j^{-\kappa }. \end{aligned}$$
2. **Domain of** $\varPsi$: the functional $\varPsi$ is defined everywhere on ${\mathcal {H}}^s$.
3. **Derivatives of** $\varPsi$: The derivative of $\varPsi$ is bounded and globally Lipschitz:
  2.19 $$\begin{aligned} \left| \left| \nabla \varPsi (x)\right| \right| _{-s} \lesssim 1,\qquad \left| \left| \nabla \varPsi (x)- \nabla \varPsi (y)\right| \right| _{-s} \lesssim \left| \left| x-y\right| \right| _{s}. \end{aligned}$$

#### Remark 2.1

The condition $\kappa > s+\frac{1}{2}$ ensures that ${\mathrm{Trace}}_{{\mathcal {H}}^s}({\mathcal {C}}_s) < \infty$. Consequently, $\pi _0$ has support in ${\mathcal {H}}^s$ ($\pi _0({\mathcal {H}}^s)=1$). $\square$

#### Example 2.1

The functional $\varPsi (x) = \sqrt{1+\left| \left| x\right| \right| _{s}^2}$ satisfies all of the above.

#### Remark 2.2

Our assumptions on the change of measure (that is, on $\varPsi$) are less general than those adopted in [14, 17] and related literature (see references therein). This is for purely technical reasons. In this paper we assume that $\varPsi$ grows linearly. If $\varPsi$ was assumed to grow quadratically, which is the case in the mentioned works, finding bounds on the moments of the chain $\{x^{k,N}\}_{k\ge 1}$ (much needed in all of the analysis) would become more involved than it already is, see Remark C.1. However, under our assumptions, the measure $\pi$ (or $\pi ^N$) is still, generically, of non-product form. $\square$

We now explore the consequences of Assumption 2.1. The proofs of the following lemmas can be found in Appendix A.

#### Lemma 2.1

Suppose that Assumption 2.1 holds. Then

1. The function ${\mathcal {C}}\nabla \varPsi (x)$ is bounded and globally Lipschitz on ${\mathcal {H}}^s$, that is
  2.20 $$\begin{aligned} \left| \left| {\mathcal {C}}\nabla \varPsi (x)\right| \right| _{s}\lesssim 1 \quad \text{ and } \quad \left| \left| {\mathcal {C}}\nabla \varPsi (x)-{\mathcal {C}}\nabla \varPsi (y)\right| \right| _{s}\lesssim \left| \left| x-y\right| \right| _{s}. \end{aligned}$$
  Therefore, the function $F(z):=-z-{\mathcal {C}}\nabla \varPsi (z)$ satisfies
  2.21 $$\begin{aligned} \left| \left| F(x) - F(y)\right| \right| _{s} \lesssim \left| \left| x-y\right| \right| _{s} \quad \text{ and } \quad \left| \left| F(x)\right| \right| _{s} \lesssim 1+ \left| \left| x\right| \right| _{s}. \end{aligned}$$
2. The function $\varPsi (x)$ is globally Lipschitz and therefore also $\varPsi ^N(x):=\varPsi ({\mathcal {P}}^N(x))$ is globally Lipschitz:
  2.22 $$\begin{aligned} \left| \varPsi ^N(y)-\varPsi ^N(x)\right| \lesssim \left| \left| y-x\right| \right| _{s}. \end{aligned}$$

Before stating the next lemma, we observe that by definition of the projection operator ${\mathcal {P}}^N$ we have that

2.23 $$\begin{aligned} \nabla \varPsi ^N={\mathcal {P}}^N\circ \nabla \varPsi \circ {\mathcal {P}}^N. \end{aligned}$$

#### Lemma 2.2

Suppose that Assumption 2.1 holds. Then the following holds for the function $\varPsi ^N$ and for its the gradient:

1. If the bounds (2.19) hold for $\varPsi$, then they hold for $\varPsi ^N$ as well:
  2.24 $$\begin{aligned} \left| \left| \nabla \varPsi ^N(x)\right| \right| _{-s}\lesssim 1,\qquad \left| \left| \nabla \varPsi ^N(x)- \nabla \varPsi ^N(y)\right| \right| _{-s} \lesssim \left| \left| x-y\right| \right| _{s}. \end{aligned}$$
2. Moreover,
  2.25 $$\begin{aligned} \left| \left| {\mathcal {C}}_N\nabla \varPsi ^N(x)\right| \right| _s\lesssim 1, \end{aligned}$$
  and
  2.26 $$\begin{aligned} \left| \left| {\mathcal {C}}_N\nabla \varPsi ^N(x)\right| \right| _{{\mathcal {C}}_N}\lesssim 1. \end{aligned}$$

We stress that in (2.24)–(2.26) the constant implied by the use of the notation “$\lesssim$” (see end of Sect. 2.1) is independent of *N*. Lastly, in what follows we will need the fact that, due assumptions on the covariance operator,

2.27 $$\begin{aligned} {\mathbb {E}}\left| \left| {\mathcal {C}}_N^{1/2} \xi ^N\right| \right| _{s}^2 \lesssim 1, \quad \hbox { uniformly in}\ N, \end{aligned}$$

where $\xi ^N:=\sum _{j=1}^N\xi _j\phi _j$ and $\xi _i {\mathop {\sim }\limits ^{{\mathcal {D}}}} {\mathcal {N}}(0,1)$ i.d.d., see [15, (2.32)] or [14, first proof of Appendix A]

## Existence and uniqueness for the limiting diffusion process

The main results of this section are Theorems 3.1, 3.2 and 3.3. Theorems 3.1 and 3.2 are concerned with establishing existence and uniqueness for Eqs. (1.10) and (1.11), respectively. Theorem 3.3 states the continuity of the Itô maps associated with Eqs.  (1.10) and (1.11). The proofs of the main results of this paper (Theorems 4.1 and 4.2) rely heavily on the continuity of such maps, as we illustrate in Sect. 5. Once Lemma 3.1 below is established, the proofs of the theorems in this section are completely analogous to the proofs of those in [14, Section 4]. For this reason, we omit them and refer the reader to [14]. In what follows, recall that the definition of the functions $\alpha _{\ell }, h_{\ell }$ and $b_{\ell }$ has been given in (1.12), (1.13) and (1.14), respectively.

### Lemma 3.1

The functions $\alpha _{\ell }(s)$, $h_{\ell }(s)$ and $\sqrt{h_{\ell }(s)}$ are positive, globally Lipschitz continuous and bounded. The function $b_{\ell }(s)$ is globally Lipschitz and it is bounded above but not below. Moreover, for any $\ell >0$, $b_{\ell }(s)$ is strictly positive for $s\in [0,1)$, strictly negative for $s>1$ and $b_{\ell }(1)=0$.

### Proof of Lemma 3.1

When $s>1$, $\alpha _{\ell }(s)=1$ while for $s\le 1$ $\alpha _{\ell }(s)$ has bounded derivative; therefore $\alpha _{\ell }(s)$ is globally Lipshitz. A similar reasoning gives the Lipshitzianity of the other functions. The further properties of $b_{\ell }$ are straightforward from the definition. $\square$

In the case of (1.11) we have the following.

### Theorem 3.1

For any initial datum $S(0) >0$, there exists a unique solution *S*(*t*) to the ODE (1.11). The solution is strictly positive for any $t>0$, it is bounded and has continuous first derivative for all $t\ge 0$. In particular

$$\begin{aligned} \lim _{t\rightarrow \infty } S(t) =1 \, \end{aligned}$$

and

3.1 $$\begin{aligned} 0\le \min \{S(0),1\}\le S(t) \le \max \{S(0), 1\} \, . \end{aligned}$$

For (1.10) we have that:

### Theorem 3.2

Let Assumption 2.1 hold and consider Eq. (1.10), where *W*(*t*) is any ${\mathcal {H}}^s$-valued ${{{\mathcal {C}}}}_s$-Brownian motion and *S*(*t*) is the solution of (1.11). Then for any initial condition $x^0\in {\mathcal {H}}^s$ and any $T>0$ there exists a unique solution of Eq. (1.10) in the space $C([0,T]; {\mathcal {H}}^s)$.

Consider the deterministic equations

3.2 $$\begin{aligned} dz(t)=[-z(t)-{\mathcal {C}}\nabla \varPsi (z(t))] h_{\ell }(S(t)) \, dt + d\zeta (t),\qquad z(0)=z^0 \end{aligned}$$

and

3.3 $$\begin{aligned} d{\mathfrak {S}}(t)=b_{\ell }({\mathfrak {S}}(t)) \, dt+ dw(t),\qquad {\mathfrak {S}}(0)={\mathfrak {S}}^0, \end{aligned}$$

where *S* is the solution of (1.11), $z^0\in {\mathcal {H}}^s$, ${\mathfrak {S}}^0\in {\mathbb {R}}$, and $\zeta$ and *w* are functions in $C([0,T];{\mathcal {H}}^s)$ and $C([0,T];{\mathbb {R}})$, respectively. Throughout the paper, we endow the spaces $C([0,T];{\mathcal {H}}^s)$ and $C([0,T];{\mathbb {R}})$ with the uniform topology. The following is the starting point of the continuous mapping arguments presented in Sect. 5.

### Theorem 3.3

Suppose that Assumption 2.1 is satisfied. Both (3.2) and (3.3) have unique solutions in $C([0,T];{\mathcal {H}}^s)$ and $C([0,T];{\mathbb {R}})$, respectively. The Itô maps

$$\begin{aligned} {\mathcal {J}}_1: {\mathcal {H}}^s\times C([0,T]; {\mathcal {H}}^s)&\longrightarrow C([0,T];{\mathcal {H}}^s) \\ (z^0,\zeta )&\longrightarrow z \end{aligned}$$

and

$$\begin{aligned} {\mathcal {J}}_2: {\mathbb {R}}_+ \times C([0,T]; {\mathbb {R}})&\longrightarrow C([0,T]; {\mathbb {R}}) \\ ({\mathfrak {S}}^0, w)&\longrightarrow {\mathfrak {S}} \end{aligned}$$

are continuous.

## Main theorems and heuristics of proofs

In order to state the main results, we first set

4.1 $$\begin{aligned} {\mathcal {H}}^s_{\cap }:=\left\{ x \in {\mathcal {H}}^s: \lim _{N \rightarrow \infty } \frac{1}{N}\sum _{i=1}^N \frac{\left| x_i \right| ^2}{ \lambda _i^2}< \infty \right\} , \end{aligned}$$

where we recall that in the above $x_i:= \left\langle x,\phi _i \right\rangle$.

### Theorem 4.1

Let Assumption 2.1 hold and let $\delta =\ell /N^{\frac{1}{2}}$. Let $x^0\in {\mathcal {H}}^s_{\cap }$ and $T>0$. Then, as $N\rightarrow \infty$, the continuous interpolant $S^{(N)}(t)$ of the sequence $\{S^{k,N}\}_{k\in {\mathbb {N}}} \subseteq {\mathbb {R}}_+$ (defined in (1.16)) and started at $S^{0,N}=\frac{1}{N}\sum _{i=1}^N \left| x_{i}^{0} \right| ^2 / \lambda _i^2$, converges in probability in $C([0,T]; {\mathbb {R}})$ to the solution *S*(*t*) of the ODE (1.11) with initial datum $S^0:=\lim _{N\rightarrow \infty }S^{0,N}$.

For the following theorem recall that the solution of (1.10) is interpreted precisely through Theorem 3.2 as a process driven by an ${\mathcal {H}}^s-$valued Brownian motion with covariance ${\mathcal {C}}_s$, and solution in $C([0,T];{\mathcal {H}}^s).$

### Theorem 4.2

Let Assumption 2.1 hold let $\delta =\ell /N^{\frac{1}{2}}$. Let $x^0\in {\mathcal {H}}^s_{\cap }$ and $T>0$. Then, as $N \rightarrow \infty$, the continuous interpolant $x^{(N)}(t)$ of the chain $\{x^{k,N}\}_{k\in {\mathbb {N}}} \subseteq {\mathcal {H}}^s$ (defined in (1.9) and (2.14), respectively) with initial state $x^{0,N}:={\mathcal {P}}^N(x^0)$, converges weakly in $C([0,T]; {\mathcal {H}}^s)$ to the solution *x*(*t*) of Eq. (1.10) with initial datum $x^0$. We recall that the time-dependent function *S*(*t*) appearing in (1.10) is the solution of the ODE (1.11), started at $S(0):= \lim _{N \rightarrow \infty } \frac{1}{N}\sum _{i=1}^N \left| x_i^{0} \right| ^2 / \lambda _i^2$.

Both Theorems 4.1 and 4.2 assume that the initial datum of the chains $x^{k,N}$ is assigned deterministically. From our proofs it will be clear that the same statements also hold for random initial data, as long as (i) $x^{0,N}$ is not drawn at random from the target measure $\pi ^N$ or from any other measure which is a change of measure from $\pi ^N$ (i.e. we need to be starting out of stationarity) and (ii) $S^{0,N}$ and $x^{0,N}$ have bounded moments (bounded uniformly in *N*) of sufficiently high order and are independent of all the other sources of noise present in the algorithm. Notice moreover that the convergence in probability of Theorem 4.1 is equivalent to weak convergence, as the limit is deterministic.

The rigorous proof of the above results is contained in Sects. 5–8. In the remainder of this section we give heuristic arguments to justify our choice of scaling $\delta \propto N^{-1/2}$ and we explain how one can formally obtain the (fluid) ODE limit (1.11) for the double sequence $S^{k,N}$ and the diffusion limit (1.10) for the chain $x^{k,N}$. We stress that the arguments of this section are only formal; therefore, we often use the notation “$\simeq$”, to mean “approximately equal”. That is, we write $A\simeq B$ when $A=B+$ “terms that are negligible” as *N* tends to infinity; we then justify these approximations, and the resulting limit theorems, in the following Sects. 5–8.

### Heuristic analysis of the acceptance probability

As observed in [17, equation (2.21)], the acceptance probability (2.10) can be expressed as

4.2 $$\begin{aligned} \alpha ^N\big (x^N,\xi ^N\big )= 1\wedge e^{Q^N\big (x^N,\xi ^N\big )}, \end{aligned}$$

where, using the notation (2.1), the function $Q^N(x,\xi )$ can be written as

4.3 $$\begin{aligned} Q^N(x^N, \xi ^N)&:= - \frac{\delta }{4} \left( \left| \left| y^N\right| \right| _{{\mathcal {C}}_N}^2 - \left| \left| x^N\right| \right| _{{\mathcal {C}}_N}^2\right) + r^N (x^N, \xi ^N) \end{aligned}$$

4.4 $$\begin{aligned}&= \left[ \frac{\delta ^2}{2}\left( \left| \left| x^N\right| \right| _{{\mathcal {C}}_N}^2- \left| \left| {\mathcal {C}}_N^{1/2}\xi ^N\right| \right| _{{\mathcal {C}}_N}^2 \right) \right] - \frac{\delta ^3}{4} \left| \left| x^N\right| \right| _{{\mathcal {C}}_N}^2 \nonumber \\&\quad -\left( \frac{\delta ^{3/2}}{\sqrt{2}} - \frac{\delta ^{5/2}}{\sqrt{2}} \right) \langle x^N, {\mathcal {C}}_N^{1/2} \xi ^N\rangle _{{\mathcal {C}}_N}+r_{\varPsi }^N (x^N, \xi ^N). \end{aligned}$$

We do not give here a complete expression for the terms $r^{N}(x^N,\xi ^N)$ and $r^N_{\varPsi }(x^N,\xi ^N)$. For the time being it is sufficient to point out that

4.5 $$\begin{aligned} r^N\big (x^N,\xi ^N\big )&:=I_2^N+ I_3^N \nonumber \\ r^N_{\varPsi }\big (x^N,\xi ^N\big )&:= r^N\big (x^N,\xi ^N\big ) + \frac{\left( \delta ^2-\delta ^3 \right) }{2} \big \langle x^N, {\mathcal {C}}_N \nabla \varPsi ^N\big (x^N\big )\big \rangle _{{\mathcal {C}}_N}\nonumber \\&\quad - \frac{\delta ^3}{4}\big \Vert {\mathcal {C}}_N \nabla \varPsi ^N\big (x^N\big ) \big \Vert _{{\mathcal {C}}_N}^2+ \frac{\delta ^{5/2}}{\sqrt{2}} \big \langle {\mathcal {C}}_N \nabla \varPsi ^N\big (x^N\big ), {\mathcal {C}}_N^{1/2}\xi ^N \big \rangle _{{\mathcal {C}}_N} \end{aligned}$$

where $I_2^N$ and $I_3^N$ will be defined in (6.10) and (6.11), respectively. Because $I_2^N$ and $I_3^N$ depend on $\varPsi$, $r^N_{\varPsi }$ contains all the terms where the functional $\varPsi$ appears; moreover $r^N_{\varPsi }$ vanishes when $\varPsi =0$. The analysis of Sect. 6 (see Lemma 6.4) will show that with our choice of scaling, $\delta = \ell / N^{1/2}$, the terms $r^N$ and $r^N_{\varPsi }$ are negligible (for *N* large). Let us now illustrate the reason behind our choice of scaling. To this end, set $\delta = \ell / N^{\zeta }$ and observe the following two simple facts:

4.6 $$\begin{aligned} S^{k,N}= \frac{1}{N}\sum _{j=1}^N \frac{\left| x^{k,N}_j\right| ^2}{\lambda _j^2}= \frac{1}{N} \left| \left| x^{k,N}\right| \right| _{{\mathcal {C}}_N}^2 \end{aligned}$$

and

4.7 $$\begin{aligned} \left| \left| {\mathcal {C}}_N^{1/2}\xi ^N\right| \right| _{{\mathcal {C}}_N}^2=\sum _{i=1}^N \left| \xi _i\right| ^2 \simeq N, \end{aligned}$$

the latter fact being true by the Law of Large Numbers. Neglecting the terms containing $\varPsi$, at step *k* of the chain we have, formally,

4.8 $$\begin{aligned} Q^N(x^{k,N}, \xi ^{k+1,N}) \simeq&\frac{\ell ^2}{2} N^{1-2\zeta } \left( S^{k,N}- 1 \right) \end{aligned}$$

4.9 $$\begin{aligned}&- \frac{\ell ^3}{4} N^{1-3\zeta } S^{k,N} - \frac{\ell ^{3/2}}{\sqrt{2}} N^{(1-3\zeta )/2} \frac{\langle x^{k,N}, {\mathcal {C}}_N^{1/2} \xi ^{k,N}\rangle _{{\mathcal {C}}_N}}{\sqrt{N}} \nonumber \\\end{aligned}$$

4.10 $$\begin{aligned}&+ \frac{\ell ^{5/2}}{\sqrt{2}} N^{(1-5\zeta )/2} \frac{\langle x^{k,N}, {\mathcal {C}}_N^{1/2} \xi ^{k,N}\rangle _{{\mathcal {C}}_N}}{\sqrt{N}}. \end{aligned}$$

The above approximation (which, we stress again, is only formal and will be made rigorous in subsequent sections) has been obtained from (4.4) by setting $\delta = \ell / N^{\zeta }$ and using (4.6) and (4.7), as follows:

4.11 $$\begin{aligned} \frac{\delta ^2}{2} \left[ \left| \left| x^N\right| \right| _{{\mathcal {C}}_N}^2- \left| \left| {\mathcal {C}}_N^{1/2}\xi ^N\right| \right| _{{\mathcal {C}}_N}^2\right]&\simeq (4.8),\\ - \delta ^3 \frac{\left| \left| x^N\right| \right| _{{\mathcal {C}}_N}^2}{4} - \frac{\delta ^{3/2}}{\sqrt{2}} \langle x^N, {\mathcal {C}}_N^{1/2} \xi ^N\rangle _{{\mathcal {C}}^N}&\simeq (4.9), \nonumber \\ + \frac{\delta ^{5/2}}{\sqrt{2}} \langle x^N, {\mathcal {C}}_N^{1/2} \xi ^N\rangle _{{\mathcal {C}}^N}&= (4.10). \nonumber \end{aligned}$$

Looking at the decomposition (4.8)–(4.10) of the function $Q^N$, we can now heuristically explain the reason why we are lead to choose $\zeta =1/2$ when we start the chain out of stationarity, as opposed to the scaling $\zeta =1/3$ when the chain is started in stationarity. This is explained in the following remark.

#### Remark 4.1

First notice that the expression (4.4) and the approximation (4.8)–(4.10) for $Q^N$ are valid both in and out of stationarity, as the first is only a consequence of the definition of the Metropolis–Hastings algorithm and the latter is implied just by the properties of $\varPsi$ and by our definitions.

- If we start the chain in stationarity, i.e. $x_0^N\sim \pi ^N$ (where $\pi ^N$ has been defined in (1.6)), then $x^{k,N} \sim \pi ^N$ for every $k \ge 0$. As we have already observed, $\pi ^N$ is absolutely continuous with respect to the Gaussian measure $\pi _0^N \sim {\mathcal {N}}(0, {\mathcal {C}}_N)$; because all the almost sure properties are preserved under this change of measure, in the stationary regime most of the estimates of interest need to be shown only for $x^N \sim \pi _0^N$. In particular if $x^N \sim \pi _0^N$ then $x^N$ can be represented as $x^N= \sum _{i=1}^N \lambda _i \rho _i \phi _i$, where $\rho _i$ are i.i.d.  ${\mathcal {N}}(0,1)$. Therefore we can use the law of large numbers and observe that $\Vert x^N\Vert _{{\mathcal {C}}^N}^2=\sum _{i=1}^N \left| \rho _{i} \right| ^2 \simeq N$.
- Suppose we want to study the algorithm in stationarity and we therefore make the choice $\zeta =1/3$. With the above point in mind, notice that if we start in stationarity then by the Law of Large numbers $N^{-1}\sum _{i=1}^N \left| \rho _{i} \right| ^2= S^{k,N}\rightarrow 1$ (as $N\rightarrow \infty$, with speed of convergence $N^{-1/2}$). Moreover, if $x^N \sim \pi _0^N$, by the Central Limit Theorem the term $\langle x^N, {\mathcal {C}}_N^{1/2} \xi ^N\rangle _{{\mathcal {C}}_N}/\sqrt{N}$ is *O*(1) and converges to a standard Gaussian. With these two observations in place we can then heuristically see that, with the choice $\zeta =1/3$ the term in (4.10) are negligible as $N\rightarrow \infty$ while the terms in (4.9) are *O*(1). The term in (4.8) can be better understood by looking at the LHS of (4.11) which, with $\zeta =1/3$ and $x^N \sim \pi _0^N$, can be rewritten as
  4.12 $$\begin{aligned} \frac{\ell ^2}{2N^{2/3}} \sum _{i=1}^N (\left| \rho _i\right| ^2- \left| \xi _i\right| ^2 ). \end{aligned}$$
  The expected value of the above expression is zero. If we apply the Central Limit Theorem to the i.i.d. sequence $\{\left| \rho _i\right| ^2- \left| \xi _i\right| ^2 \}_i$, (4.12) shows that (4.8) is $O(N^{1/2-2/3})$ and therefore negligible as $N \rightarrow \infty$. In conclusion, in the stationary case the only *O*(1) terms are those in (4.9); therefore one has the heuristic approximation
  $$\begin{aligned} Q^N(x,\xi ) \sim {\mathcal {N}} \left( -\frac{\ell ^3}{4}, \frac{\ell ^3}{2}\right) . \end{aligned}$$
  For more details on the stationary case see [17].
- If instead we start out of stationarity the choice $\zeta =1/3$ is problematic. Indeed in [6, Lemma 3] the authors study the MALA algorithm to sample from an *N*-dimensional isotropic Gaussian and show that if the algorithm is started at a point $x^0$ such that $S(0) <1$, then the acceptance probability degenerates to zero. Therefore, the algorithm stays stuck in its initial state and never proceeds to the next move, see [6, Figure 2] (to be more precise, as *N* increases the algorithm will take longer and longer to get unstuck from its initial state; in the limit, it will never move with probability 1). Therefore the choice $\zeta =1/3$ cannot be the optimal one (at least not irrespective of the initial state of the chain) if we start out of stationarity. This is still the case in our context and one can heuristically see that the root of the problem lies in the term (4.8). Indeed if out of stationarity we still choose $\zeta =1/3$ then, like before, (4.9) is still order one and (4.10) is still negligible. However, looking at (4.8), if $x^0$ is such that $S(0)<1$ then, when $k=0$, (4.8) tends to minus infinity; recalling (4.2), this implies that the acceptance probability of the first move tends to zero. To overcome this issue and make $Q^N$ of order one (irrespective of the initial datum) so that the acceptance probability is of order one and does not degenerate to 0 or 1 when $N \rightarrow \infty$, we take $\zeta =1/2$; in this way the terms in (4.8) are *O*(1), all the others are small. Therefore, the intuition leading the analysis of the non-stationary regime hinges on the fact that, with our scaling,
  4.13 $$\begin{aligned} Q^N(x^{k,N}, \xi ^{k,N}) \simeq \frac{\ell ^2}{2}(S^{k,N} -1); \end{aligned}$$
  hence
  4.14 $$\begin{aligned} \alpha ^N(x^{k,N}, \xi ^{k,N}) = (1 \wedge e^{Q^N(x^{k,N}, \xi ^{k,N})}) \simeq \alpha _{\ell }\big (S^{k,N}\big ), \end{aligned}$$
  where the function $\alpha _{\ell }$ on the RHS of (4.14) is the one defined in (1.12). The approximation (4.13) is made rigorous in Lemma 6.4, while (4.14) is formalized in Sect. 6.1 (see in particular Proposition 6.1).
- Finally, we mention for completeness that, by arguing similarly to what we have done so far, if $\zeta < 1/2$ then the acceptance probability of the first move tends to zero when $S(0)<1$. If $\zeta >1/2$ then $Q^N \rightarrow 0$, so the acceptance probability tends to one; however the size of the moves is small and the algorithm explores the phase space slowly.

#### Remark 4.2

Notice that in stationarity the function $Q^N$ is, to leading order, independent of $\xi$; that is, $Q^N$ and $\xi$ are asymptotically independent (see [17, Lemma 4.5]). This can be intuitively explained because in stationarity the leading order term in the expression for $Q^N$ is the term with $\delta ^3 \Vert x\Vert ^2$. We will show that also out of stationarity $Q^N$ and $\xi$ are asymptotically independent. In this case such an asymptotic independence can, roughly speaking, be motivated by the approximation (4.13), (as the interpolation of the chain $S^{k,N}$ converges to a deterministic limit). The asymptotic correlation of $Q^N$ and the noise $\xi$ is analysed in Lemma 6.5.

#### Remark 4.3

When one employs the more general proposal (1.18), assuming $\varPsi \equiv 0$, the expression for $Q^N$ becomes

$$\begin{aligned} Q^N\big (x^{k,N}, y^{k,N}\big ) = -\frac{\delta }{4} (1-2\theta ) \left( \Vert x^{k,N}\Vert _{{\mathcal {C}}^N}^2 - \Vert y^{k,N}\Vert _{{\mathcal {C}}^N}^2 \right) . \end{aligned}$$

So, if $\theta =1/2$, the acceptance probability would be exactly one (for every *N*), i.e. the algorithm would be sampling exactly from the prior hence there is no need of rescaling $\delta$ with *N*.

### Heuristic derivation of the weak limit of $S^{k,N}$

Let *Y* be any function of the random variables $\xi ^{k,N}$ and $U^{k,N}$ (introduced in Sect. 2.2), for example the chain $x^{k,N}$ itself. Here and throughout the paper we use ${\mathbb {E}}_{x^0}\left[ Y\right]$ to denote the expected value of *Y* with respect to the law of the variables $\xi ^{k,N}$’s and $U^{k,N}$’s, with the initial state $x_0$ of the chain given deterministically; in other words, ${\mathbb {E}}_{x^0}(Y)$ denotes expectation with respect to all the sources of randomness present in *Y*. We will use the notation ${\mathbb {E}}_k \left[ Y\right]$ for the conditional expectation of *Y* given $x^{k,N}$, ${\mathbb {E}}_k \left[ Y\right] :={\mathbb {E}}_{x^0}\left[ Y\left| x^{k,N}\right. \right]$ (we should really be writing ${\mathbb {E}}_k^N$ in place of ${\mathbb {E}}_k$, but to improve readability we will omit the further index *N*). Let us now decompose the chain $S^{k,N}$ into its drift and martingale parts:

4.15 $$\begin{aligned} S^{k+1,N}=S^{k,N}+\frac{1}{\sqrt{N}} b_{\ell }^{k,N}+ \frac{1}{N^{1/4}}D^{k,N}, \end{aligned}$$

where

4.16 $$\begin{aligned} b_{\ell }^{k,N}:=\sqrt{N}{\mathbb {E}}_k [S^{k+1,N}-S^{k,N}] \end{aligned}$$

and

4.17 $$\begin{aligned} D^{k,N}:= N^{1/4}\left[ S^{k+1,N}-S^{k,N} - \frac{1}{\sqrt{N}}b_{\ell }^{k,N}\big (x^{k,N}\big )\right] . \end{aligned}$$

In this subsection we give the heuristics which underly the proof, given in subsequent sections, that the approximate drift $b_{\ell }^{k,N}= b_{\ell }^{k,N}\big (x^{k,N}\big )$ converges to $b_{\ell }(S^{k,N})$,^2^ where $b_{\ell }$ is the drift of (1.11), while the approximate diffusion $D^{k,N}$ tends to zero. This formally gives the result of Theorem 4.1. Let us formally argue such a convergence result. By (4.6) and (2.12),

4.18 $$\begin{aligned} S^{k+1,N}= \frac{1}{N} \sum _{j=1}^N \frac{\left| x^{k+1,N}_j\right| ^2}{\lambda _j^2} = \frac{1}{N} \left( \gamma ^{k,N}\left| \left| y^{k,N}\right| \right| _{{\mathcal {C}}_N}^2 + (1-\gamma ^{k,N})\left| \left| x^{k,N}\right| \right| _{{\mathcal {C}}_N}^2 \right) . \end{aligned}$$

Therefore, again by (4.6),

4.19 $$\begin{aligned} b_{\ell }^{k,N}=\sqrt{N} {\mathbb {E}}_k \big [S^{k+1,N} -S^{k,N}\big ]&= \frac{1}{\sqrt{N}} {\mathbb {E}}_k \left[ \gamma ^{k,N}\left( \left| \left| y^{k,N}\right| \right| _{{\mathcal {C}}_N}^2 -\left| \left| x^{k,N}\right| \right| _{{\mathcal {C}}_N}^2 \right) \right] \nonumber \\&= \frac{1}{\sqrt{N}} {\mathbb {E}}_k \left[ \big (1 \wedge e^{Q^N\big (x^{k,N},y^{k,N}\big )}\big ) \left( \left| \left| y^{k,N}\right| \right| _{{\mathcal {C}}_N}^2 \right. \right. \nonumber \\&\quad \left. \left. -\left| \left| x^{k,N}\right| \right| _{{\mathcal {C}}_N}^2 \right) \right] , \end{aligned}$$

where the second equality is a consequence of the definition of $\gamma ^{k,N}$ (with a reasoning, completely analogous to the one in [14, last proof of Appendix A], see also (4.24). Using (4.3) (with $\delta =\ell /\sqrt{N}$), the fact that $r^N$ is negligible and the approximation (4.13), the above gives

$$\begin{aligned} b_{\ell }^{k,N}=\sqrt{N} {\mathbb {E}}_k [S^{k+1,N} -S^{k,N}] \simeq - \frac{4}{\ell } \left( 1 \wedge e^{\ell ^2 \big (S^{k,N}-1\big )/2} \right) \frac{\ell ^2}{2} \big (S^{k,N}-1\big ) = b_{\ell }\big (S^{k,N}\big ). \end{aligned}$$

The above approximation is made rigorous in Lemma 7.5. As for the diffusion coefficient, it is easy to check (see proof of Lemma 7.2) that

$$\begin{aligned} N {\mathbb {E}}_k [S^{k+1,N} -S^{k,N}]^2 <\infty . \end{aligned}$$

Hence the approximate diffusion tends to zero and one can formally deduce that (the interpolant of) $S^{k,N}$ converges to the ODE limit (1.11).

### Heuristic analysis of the limit of the chain $x^{k,N}$. 

The drift-martingale decomposition of the chain $x^{k,N}$ is as follows:

4.20 $$\begin{aligned} x^{k+1,N}=x^{k,N}+\frac{1}{N^{1/2}}\varTheta ^{k,N}+\frac{1}{N^{1/4}}L^{k,N}\end{aligned}$$

where $\varTheta ^{k,N}=\varTheta ^{k,N}\big (x^{k,N}\big )$ is the approximate drift

4.21 $$\begin{aligned} \varTheta ^{k,N}:=\sqrt{N} {\mathbb {E}}_k \left[ x^{k+1,N}-x^{k,N}\right] \end{aligned}$$

and

4.22 $$\begin{aligned} L^{k,N}:=N^{1/4} \left[ x^{k+1,N}- x^{k,N} - \frac{1}{\sqrt{N}} \varTheta ^{k,N}\big (x^{k,N}\big ) \right] \end{aligned}$$

is the approximate diffusion. In what follows we will use the notation $\varTheta (x,S)$ for the drift of Eq. (1.10), i.e.

4.23 $$\begin{aligned} \varTheta (x, S)= F(x)h_{\ell }(S), \quad (x, S) \in {\mathcal {H}}^s\times {\mathbb {R}}, \end{aligned}$$

with *F*(*x*) defined in Lemma 2.1. Again, we want to formally argue that the approximate drift $\varTheta ^{k,N}\big (x^{k,N}\big )$ tends to $\varTheta (x^{k,N}, S^{k,N})$^3^ and the approximate diffusion $L^{k,N}$ tends to the diffusion coefficient of Eq. (1.10).

#### Approximate drift

As a preliminary consideration, observe that

4.24 $$\begin{aligned} {\mathbb {E}}_k \left( \gamma ^{k,N}{\mathcal {C}}_N^{1/2} \xi ^{k,N}\right) = {\mathbb {E}}_k \left( \left( 1 \wedge e^{Q^N(x^{k,N}, \xi ^{k,N})} \right) {\mathcal {C}}_N^{1/2} \xi ^{k,N}\right) , \end{aligned}$$

see [14, equation (5.14)]. This fact will be used throughout the paper, often without mention. Coming to the chain $x^{k,N}$, a direct calculation based on (2.8) and on (2.12) gives

4.25 $$\begin{aligned} x^{k+1,N} - x^{k,N} = - \gamma ^{k,N}\delta \big (x^{k,N} + {\mathcal {C}}_N \nabla \varPsi ^N\big (x^{k,N}\big )\big ) + \gamma ^{k,N}\sqrt{2 \delta } {\mathcal {C}}_N^{1/2} \xi ^{k,N}.\quad \end{aligned}$$

Therefore, with the choice $\delta = \ell /\sqrt{N}$, we have

4.26 $$\begin{aligned} \varTheta ^{k,N}&=\sqrt{N}{\mathbb {E}}_k \big [x^{k+1,N} -x^{k,N}\big ] \nonumber \\&= -\ell {\mathbb {E}}_k \left[ \big (1 \wedge e^{Q^N(x^{k,N},\xi ^{k,N})}\big ) \big (x^{k,N}+ {\mathcal {C}}_N\nabla \varPsi ^N\big (x^{k,N}\big )\big ) \right] \nonumber \\&\quad +{N^{1/4}} \sqrt{2\ell } {\mathbb {E}}_k \left[ \big (1 \wedge e^{Q^N(x^{k,N},\xi ^{k,N})}\big ) {\mathcal {C}}_N^{1/2} \, \xi ^{k,N}\right] \end{aligned}$$

The addend in (4.26) is asymptotically small (see Lemma 6.5 and notice that this addend would just be zero if $Q^N$ and $\xi ^{k,N}$ were uncorrelated); hence, using the heuristic approximations (4.13) and (4.14),

4.27 $$\begin{aligned} \varTheta ^{k,N}=\sqrt{N}{\mathbb {E}}_k [x^{k+1,N} -x^{k,N}]&\simeq - \ell \alpha _{\ell }\big (S^{k,N} \big )\big (x^{k,N}+ {\mathcal {C}}_N\nabla \varPsi ^N\big (x^{k,N}\big )\big )\nonumber \\&{\mathop {=}\limits ^{(1.13)}} - h_{\ell }\big (S^{k,N}\big ) \big (x^{k,N}+ {\mathcal {C}}_N\nabla \varPsi ^N\big (x^{k,N}\big )\big );\nonumber \\ \end{aligned}$$

the right hand side of the above is precisely the limiting drift $\varTheta (x^{k,N},S^{k,N})$.

#### Approximate diffusion

We now look at the approximate diffusion of the chain $x^{k,N}$:

$$\begin{aligned} L^{k,N}:= N^{1/4} (x^{k+1,N}-x^{k,N}-{\mathbb {E}}_k(x^{k+1,N}-x^{k,N}) ). \end{aligned}$$

By definition,

4.28 $$\begin{aligned} {\mathbb {E}}_k\left| \left| L^{k,N}\right| \right| _{s}^2&= \sqrt{N}{\mathbb {E}}_k \left| \left| x^{k+1,N}-x^{k,N}\right| \right| _{s}^2 - \sqrt{N}\left| \left| {\mathbb {E}}_k\left( x^{k+1,N}-x^{k,N}\right) \right| \right| _{s}^2. \end{aligned}$$

By (4.27) the second addend in the above is asymptotically small. Therefore

$$\begin{aligned} {\mathbb {E}}_k\left| \left| L^{k,N}\right| \right| _{s}^2&\simeq \sqrt{N}{\mathbb {E}}_k \left| \left| x^{k+1,N}-x^{k,N}\right| \right| _{s}^2\\&{\mathop {\simeq }\limits ^{(2.12), (4.25)}} {2\ell } {\mathbb {E}}_k \left| \left| \gamma ^{k,N}{\mathcal {C}}_N^{1/2} \xi ^{k,N}\right| \right| _{s}^2\\&= {2\ell }{\mathbb {E}}_k\sum _{j=1}^N j^{2s}\lambda _j^2\left( 1 \wedge e^{Q^N(x^{k,N},\xi ^{k,N})} \right) \left| \xi ^{k,N}_j\right| ^2. \end{aligned}$$

The above quantity is carefully studied in Lemma 6.6. However, intuitively, the heuristic approximation (4.14) (and the asymptotic independence of $Q^N$ and $\xi$ that (4.14) is a manifestation of) suffices to formally derive the limiting diffusion coefficient [i.e. the diffusion coefficient of (1.10)]:

$$\begin{aligned} {\mathbb {E}}_k\left| \left| L^{k,N}\right| \right| _{s}^2&\simeq 2\ell \sum _{j=1}^N j^{2s}\lambda _j^2 {\mathbb {E}}_k \left[ \big (1 \wedge e^{Q^N\big (x^{k,N},y^{k,N}\big )} \big )\left| \xi _j^{k,N}\right| ^2\right] \\&\simeq 2\ell \sum _{j=1}^N j^{2s}\lambda _j^2 {\mathbb {E}}_k \left[ \big (1 \wedge e^{\ell ^2\big (S^{k,N}-1\big )/2} \big )\left| \xi _j^{k,N}\right| ^2\right] \\&\simeq 2\ell \sum _{j=1}^N j^{2s}\lambda _j^2 \big (1 \wedge e^{\ell ^2\big (S^{k,N}-1\big )/2} \big )\\&\simeq 2\ell \, {\mathrm{Trace}}({\mathcal {C}}_s)\alpha _{\ell }\big (S^{k,N}\big ){\mathop {=}\limits ^{(1.13)}}2{\mathrm{Trace}}({\mathcal {C}}_s)\,h_{\ell }\big (S^{k,N}\big ). \end{aligned}$$

## Continuous mapping argument

In this section we outline the argument which underlies the proofs of our main results. In particular, the proofs of Theorems 4.1 and 4.2 hinge on the continuous mapping arguments that we illustrate in the following Sects. 5.1 and 5.2, respectively. The details of the proofs are deferred to the next three sections: Sect. 6 contains some preliminary results that we employ in both proofs, in Sect. 7 contains the the proof of Theorem 4.1 and Sect. 8 that of Theorem 4.2.

### Continuous mapping argument for (3.3)

Let us recall the definition of the chain $\{S^{k,N}\}_{k\in {\mathbb {N}}}$ and of its continuous interpolant $S^{(N)}$, introduced in (1.15) and (1.16), respectively. From the definition (1.16) of the interpolated process and the drift-martingale decomposition (4.15) of the chain $\{S^{k,N}\}_{k\in {\mathbb {N}}}$ we have that for any $t \in [t_k, t_{k+1})$,

$$\begin{aligned} S^{(N)}(t)&= (N^{1/2}t-k) \left[ S^{k,N}+\frac{1}{\sqrt{N}} b_{\ell }^{k,N}+ \frac{1}{N^{1/4}}D^{k,N}\right] + (k+1-tN^{1/2}) S^{k,N} \\&= S^{k,N} +(t-t_k) b_{\ell }^{k,N} + N^{1/4} (t-t_k) D^{k,N}. \end{aligned}$$

Iterating the above we obtain

$$\begin{aligned} S^{(N)}(t)&=S^{0,N} + (t-t_k) b_{\ell }^{k,N}+\frac{1}{\sqrt{N}} \sum _{j=0}^{k-1}b_{\ell }^{j,N}+ {w^N(t)}, \end{aligned}$$

where

5.1 $$\begin{aligned} w^N(t):=\frac{1}{N^{1/4}}\sum _{j=0}^{k-1}D^{j,N}+N^{1/4}(t-t_k) D^{k,N}\quad t_k\le t <t_{k+1}. \end{aligned}$$

The expression for $S^{(N)}(t)$ can then be rewritten as

5.2 $$\begin{aligned} S^{(N)}(t) = S^{0,N}+\int _0^t b_{\ell }(S^{(N)}(v)) dv+ {\hat{w}}^N(t), \end{aligned}$$

having set

5.3 $$\begin{aligned} {\hat{w}}^N(t):=e^N(t)+w^N(t), \end{aligned}$$

with

5.4 $$\begin{aligned} e^N(t):=(t-t_k) b_{\ell }^{k,N}+\frac{1}{\sqrt{N}} \sum _{j=0}^{k-1}b_{\ell }^{j,N}-\int _0^t b_{\ell }(S^{(N)}(v)) dv. \end{aligned}$$

Equation (5.2) shows that

$$\begin{aligned} S^{(N)}={\mathcal {J}}_2(S^{0,N},{\hat{w}}^N), \end{aligned}$$

where ${\mathcal {J}}_2$ is the Itô map defined in the statement of Theorem 3.3. By the continuity of the map ${\mathcal {J}}_2$, if we show that ${\hat{w}}^N$ converges in probability in $C([0,T]; {\mathbb {R}})$ to zero, then $S^{(N)}(t)$ converges in probability to the solution of the ODE (1.11). We prove convergence of ${\hat{w}}^N$ to zero in Sect. 7. In view of (5.3), we show the convergence in probability of ${\hat{w}}^N$ to zero by proving that both $e^N$ (Lemma 7.1) and $w^N$ (Lemma 7.2) converge in $L_2(\varOmega ; C([0,T]; {\mathbb {R}}))$ to zero. Because $\{S^{0,N}\}_{N\in {\mathbb {N}}}$ is a deterministic sequence that converges to $S^0$, we then have that $(S^{0,N},{\hat{w}}^N)$ converges in probability to $(S^0,0)$.

### Continuous mapping argument for (3.2)

We now consider the chain $\{x^{k,N}\}_{k\in {\mathbb {N}}}\subseteq {\mathcal {H}}^s$, defined in (2.14). We act analogously to what we have done for the chain $\{S^{k,N}\}_{k\in {\mathbb {N}}}$. So we start by recalling the definition of the continuous interpolant $x^{(N)}$, Eq. (1.9) and the notation introduced at the beginning of Sect. 4.3. An argument analogous to the one used to derive (5.2) shows that for any $t\in [t_k,t_{k+1})$

5.5 $$\begin{aligned} x^{(N)}(t)&=x^{0,N}+ (t-t_k) \varTheta ^{k,N}+ \frac{1}{\sqrt{N}}\sum _{j=0}^k\varTheta ^{j,N}+{\eta ^N(t)}\nonumber \\&= x^{0,N}+\int _0^t \varTheta (x^{(N)}(v),S(v)) dv+ {\hat{\eta }}^N(t), \end{aligned}$$

where

5.6 $$\begin{aligned} {\hat{\eta }}^N(t)&:=d^N(t)+\upsilon ^N(t)+\eta ^N(t), \end{aligned}$$

5.7 $$\begin{aligned} {\eta }^N(t)&:={N^{1/4}(t-t_k)L^{k,N}+\frac{1}{N^{1/4}} \sum _{j=1}^{k-1}L^{j,N}}, \end{aligned}$$

and

5.8 $$\begin{aligned} d^N(t)&:= (t-t_k) \varTheta ^{k,N}+ \frac{1}{\sqrt{N}}\sum _{j=0}^{k-1}\varTheta ^{j,N} - \int _0^t \varTheta (x^{(N)}(v),S^{(N)}(v))dv, \end{aligned}$$

5.9 $$\begin{aligned} \upsilon ^N(t)&:=\int _0^t \left[ \varTheta (x^{(N)}(v),S^{(N)}(v))- \varTheta (x^{(N)}(v),S(v))\right] dv. \end{aligned}$$

Equation (5.5) implies that

5.10 $$\begin{aligned} x^{(N)}={\mathcal {J}}_1(x^{0,N},{\hat{\eta }}^N), \end{aligned}$$

where ${\mathcal {J}}_1$ is Itô map defined in the statement of Theorem 3.3. In Sect. 8 we prove that ${\hat{\eta }}^N$ converges weakly in $C([0,T];{\mathcal {H}}^s)$ to the process $\eta$, where the process $\eta$ is the diffusion part of Eq. (1.10), i.e.

5.11 $$\begin{aligned} \eta (t):=\int _0^t \sqrt{2h_{\ell }(S(v))} dW_v, \end{aligned}$$

with $W_v$ a ${\mathcal {H}}^s$-valued ${\mathcal {C}}_s$-Brownian motion. Looking at (5.6), we prove the weak convergence of ${\hat{\eta }}^N$ to $\eta$ by the following steps:

1. We prove that $d^N$ converges in $L_2(\varOmega ; C([0,T]; {\mathcal {H}}^s))$ to zero (Lemma 8.1);
2. using the convergence in probability (in $C([0,T]; {\mathbb {R}})$) of $S^{(N)}$ to *S*, we show convergence in probability (in $C([0,T]; {\mathcal {H}}^s)$) of $\upsilon ^N$ to zero (Lemma 8.2);
3. we show that $\eta ^N$ converges in weakly in $C([0,T]; {\mathcal {H}}^s)$ to the process $\eta$, defined in (5.11) (Lemma 8.3).

Because $\{x^{0,N}\}_{N\in {\mathbb {N}}}$ is a deterministic sequence that converges to $x^0$, the above three steps (and Slutsky’s Theorem) imply that $(x^{0,N},{\hat{\eta }}^N)$ converges weakly to $(x^0,\eta )$. Now observe that $x(t)={\mathcal {J}}_1(x^0, \eta (t))$, where *x*(*t*) is the solution of the SDE (1.10). The continuity of the map ${\mathcal {J}}_1$ (Theorem 3.3), (5.10) and the Continuous Mapping Theorem then imply that the sequence $\{x^{(N)}\}_{N\in {\mathbb {N}}}$ converges weakly to the solution of the SDE (1.10), thus establishing Theorem 4.2.

## Preliminary estimates and analysis of the acceptance probability

This section gathers several technical results. In Lemma 6.1 we study the size of the jumps of the chain. Lemma 6.2 contains uniform bounds on the moments of the chains $\{x^{k,N}\}_{k\in {\mathbb {N}}}$ and $\{S^{k,N}\}_{k\in {\mathbb {N}}}$, much needed in Sects. 7 and 8. In Section 6.1 we detail the analysis of the acceptance probability. This allows us to quantify the correlations between $\gamma ^{k,N}$ and the noise $\xi ^{k,N}$, Sect. 6.2. Throughout the paper, when referring to the function $Q^N$ defined in (4.3), we use interchangeably the notation $Q^N(x^{k,N}, y^{k,N})$ and $Q^N(x^{k,N}, \xi ^{k,N})$ (as we have already remarked, given $x^{k,N}$, the proposal $y^{k,N}$ is only a function of $\xi ^{k,N}$).

### Lemma 6.1

Let $q\ge 1/2$ be a real number. Under Assumption 2.1 the following holds:

6.1 $$\begin{aligned} {\mathbb {E}}_k{\left| \left| y^{k,N}-x^{k,N}\right| \right| _{s}^{2q}}\lesssim \frac{1}{N^{q/2}}\left( 1+\left| \left| x^{k,N}\right| \right| _{s}^{2q}\right) \end{aligned}$$

and

6.2 $$\begin{aligned} {\mathbb {E}}_k{\left| \left| y^{k,N}-x^{k,N}\right| \right| _{{\mathcal {C}}_N}^{2q}}\lesssim \big (S^{k,N}\big )^q+N^{q/2}. \end{aligned}$$

Therefore,

6.3 $$\begin{aligned} {\mathbb {E}}_k{\left| \left| x^{k+1,N}-x^{k,N}\right| \right| _{s}^{2q}}\lesssim \frac{1}{N^{q/2}}\left( 1+\left| \left| x^{k,N}\right| \right| _{s}^{2q}\right) , \end{aligned}$$

and

6.4 $$\begin{aligned} {\mathbb {E}}_k{\left| \left| x^{k+1,N}-x^{k,N}\right| \right| _{{\mathcal {C}}_N}^{2q}}\lesssim \big (S^{k,N}\big )^q+N^{q/2}. \end{aligned}$$

### Proof of Lemma 6.1

By definition of the proposal $y^{k,N}$, Eq. (2.8),

$$\begin{aligned} \left| \left| y^{k,N}-x^{k,N}\right| \right| _{s}^{2q} =&\, \left| \left| \delta \big (x^{k,N}+{\mathcal {C}}_N\nabla \varPsi ^N\big (x^{k,N}\big )\big )+\sqrt{2\delta } {\mathcal {C}}_N^{1/2}\xi ^{k,N}\right| \right| _{s}^{2q}\\ \lesssim&\,\frac{1}{N^q}\left( \left| \left| x^{k,N}\right| \right| _{s}^{2q} +\left| \left| {\mathcal {C}}_N\nabla \varPsi ^N\big (x^{k,N}\big )\right| \right| _{s}^{2q}\right) \nonumber \\&+\frac{1}{N^{q/2}} \left| \left| {\mathcal {C}}_N^{1/2}\xi ^{k,N}\right| \right| _{s}^{2q}. \end{aligned}$$

Thus, using (2.25) and (2.27), we have

$$\begin{aligned} {\mathbb {E}}_k\left| \left| y^{k,N}-x^{k,N}\right| \right| _{s}^{2q}&\lesssim \frac{1}{N^q} \left( 1+\left| \left| x^{k,N}\right| \right| _{s}^{2q}\right) +\frac{1}{N^{q/2}}\\&\lesssim \frac{1}{N^{q/2}}\left( 1+\left| \left| x^{k,N}\right| \right| _{s}^{2q}\right) , \end{aligned}$$

which proves (6.1). Equation (6.2) follows similarly:

$$\begin{aligned} {\mathbb {E}}_k{\left| \left| y^{k,N}-x^{k,N}\right| \right| _{{\mathcal {C}}_N}^{2q}}\lesssim&\, \frac{1}{N^q}\left( \left| \left| x^{k,N}\right| \right| _{{\mathcal {C}}_N}^{2q}+ \left| \left| {\mathcal {C}}_N\nabla \varPsi ^N\big (x^{k,N}\big )\right| \right| _{{\mathcal {C}}_N}^{2q}\right) \\&+\frac{1}{N^{q/2}}{\mathbb {E}}_k \left| \left| {\mathcal {C}}_N^{1/2}\xi ^{k,N}\right| \right| _{{\mathcal {C}}_N}^{2q}. \end{aligned}$$

Since $\left| \left| {\mathcal {C}}_N^{1/2}\xi ^{k,N}\right| \right| _{{\mathcal {C}}_N}^{2}=\sum _{j=1}^N(\xi ^{k,N}_j)^2$ has chi-squared law, applying Stirling’s formula for the Gamma function $\varGamma :{\mathbb {R}}\rightarrow {\mathbb {R}}$ we obtain

6.5 $$\begin{aligned} {\mathbb {E}}_k\left| \left| {\mathcal {C}}_N^{1/2}\xi ^{k,N}\right| \right| _{{\mathcal {C}}_N}^{2q}\lesssim \frac{\varGamma (q+N/2)}{\varGamma (N/2)}\lesssim N^q. \end{aligned}$$

Hence, using (2.26), the desired bound follows. Finally, recalling the definition of the chain, Eq. (2.12), the bounds (6.3) and (6.4) are clearly a consequence of (6.1) and (6.2), respectively, since either $x^{k+1,N}=y^{k,N}$ (if the proposed move is accepted) or $x^{k+1,N}=x^{k,N}$ (if the move is rejected). $\square$

### Lemma 6.2

If Assumption 2.1 holds, then, for every $q\ge 1$, we have

6.6 $$\begin{aligned} {\mathbb {E}}_{x^0}\big (S^{k,N}\big )^q&\lesssim 1 \end{aligned}$$

6.7 $$\begin{aligned} {\mathbb {E}}_{x^0}{\left| \left| x^{k,N}\right| \right| _{s}^q}&\lesssim 1, \end{aligned}$$

uniformly over $N \in {\mathbb {N}}$ and $k \in \{0, 1 ,\ldots ,[T\sqrt{N}]\}$.

### Proof of Lemma 6.2

The proof of this lemma can be found in Appendix C. $\square$

### Acceptance probability

The main result of this section is Proposition 6.1, which we obtain as a consequence of Lemma 6.3 (below) and Lemma 6.2. Proposition 6.1 formalizes the heuristic approximation (4.14).

#### Lemma 6.3

(Acceptance probability) Let Assumption 2.1 hold and recall the Definitions (4.2) and (1.12). Then the following holds:

$$\begin{aligned} {\mathbb {E}}_k \left| \alpha ^N(x^{k,N},\xi ^{k,N})-\alpha _\ell \big (S^{k,N}\big )\right| ^{2}\lesssim \frac{1+\big (S^{k,N}\big )^2+\left| \left| x^{k,N}\right| \right| _{s}^2}{\sqrt{N}}. \end{aligned}$$

Before proving Lemma 6.3, we state Proposition 6.1.

#### Proposition 6.1

If Assumption 2.1 holds then

$$\begin{aligned} \lim _{N\rightarrow \infty }{\mathbb {E}}_{x^0}{\left| \alpha ^N(x^{k,N},y^{k,N})-\alpha _\ell \big (S^{k,N}\big )\right| ^{2}}=0. \end{aligned}$$

#### Proof

This is a corollary of Lemmas 6.3 and 6.2. $\square$

#### Proof of Lemma 6.3

The function $z\mapsto 1\wedge e^z$ on ${\mathbb {R}}$ is globally Lipschitz with Lipschitz constant 1. Therefore, by (1.12) and (4.2),

$$\begin{aligned} {\mathbb {E}}_k \left| \alpha ^N(x^{k,N},y^{k,N})-\alpha _\ell \big (S^{k,N}\big )\right| ^{2}\le {\mathbb {E}}_k \left| Q^N(x^{k,N},y^{k,N})-\frac{\ell ^2\big (S^{k,N}-1\big )}{2}\right| ^{2}. \end{aligned}$$

The result is now a consequence of (6.15) below. $\square$

To analyse the acceptance probability it is convenient to decompose $Q^N$ as follows:

6.8 $$\begin{aligned} Q^N\big (x^N,y^N\big )=I_1^N\big (x^N,y^N\big )+I_2^N\big (x^N,y^N\big )+I_3^N\big (x^N,y^N\big ) \end{aligned}$$

where

6.9 $$\begin{aligned} I_1^N\big (x^N,y^N\big )&:=-\frac{1}{2}\left[ \left| \left| y^N\right| \right| _{{\mathcal {C}}_N}^2 -\left| \left| x^N\right| \right| _{{\mathcal {C}}_N}^2\right] \nonumber \\&\quad -\frac{1}{4\delta } \left[ \left| \left| x^N-(1-\delta )y^N\right| \right| _{{\mathcal {C}}_N}^2-\left| \left| y^N-(1-\delta )x^N\right| \right| _{{\mathcal {C}}_N}^2\right] \nonumber \\&=-\frac{\delta }{4}\left( \left| \left| y^N\right| \right| _{{\mathcal {C}}_N}^2-\left| \left| x^N\right| \right| _{{\mathcal {C}}_N}^2\right) , \end{aligned}$$

6.10 $$\begin{aligned} I_2^N\big (x^N,y^N\big )&:=-\frac{1}{2}\left[ \left\langle x^N-(1-\delta )y^N,{\mathcal {C}}_N\nabla \varPsi ^N\big (y^N\big ) \right\rangle _{{\mathcal {C}}_N}\right. \nonumber \\&\quad \left. - \left\langle y^N-(1-\delta )x^N,{\mathcal {C}}_N\nabla \varPsi ^N\big (x^N\big ) \right\rangle _{{\mathcal {C}}_N}\right] \nonumber \\&\quad -\big (\varPsi ^N\big (y^N\big )-\varPsi ^N\big (x^N\big )\big ), \end{aligned}$$

6.11 $$\begin{aligned} I_3^N\big (x^N,y^N\big )&:=-\frac{\delta }{4}\left[ \left| \left| {\mathcal {C}}_N\nabla \varPsi ^N\big (y^N\big )\right| \right| _{{\mathcal {C}}_N}^2 -\left| \left| {\mathcal {C}}_N\nabla \varPsi ^N\big (x^N\big )\right| \right| _{{\mathcal {C}}_N}^2\right] . \end{aligned}$$

#### Lemma 6.4

Let Assumption 2.1 hold. With the notation introduced above, we have:

6.12 $$\begin{aligned} {\mathbb {E}}_k \left| I_1^N\big (x^{k,N},y^{k,N}\big )-\frac{\ell ^2\big (S^{k,N}-1\big )}{2}\right| ^2&\lesssim \frac{\left| \left| x^{k,N}\right| \right| _{s}^2}{N^2}+\frac{\big (S^{k,N}\big )^2}{\sqrt{N}}+\frac{1}{N} \end{aligned}$$

6.13 $$\begin{aligned} {\mathbb {E}}_k \left| I_2^N\big (x^{k,N},y^{k,N}\big )\right| ^2&\lesssim \frac{1+\left| \left| x^{k,N}\right| \right| _{s}^2}{\sqrt{N}} \end{aligned}$$

6.14 $$\begin{aligned} {\mathbb {E}}_k \left| I_3^N\big (x^{k,N},y^{k,N}\big )\right| ^2&\lesssim \frac{1}{N}. \end{aligned}$$

Therefore,

6.15 $$\begin{aligned} {\mathbb {E}}_k \left| Q^N\big (x^{k,N},y^{k,N}\big )-\frac{\ell ^2\big (S^{k,N}-1\big )}{2}\right| ^{2}\lesssim \frac{1+\big (S^{k,N}\big )^2+\left| \left| x^{k,N}\right| \right| _{s}^2}{\sqrt{N}}. \end{aligned}$$

#### Proof of Lemma 6.4

We consecutively prove the three bounds in the statement.

- Proof of (6.12). Using (2.8), we rewrite $I_1^N$ as
  $$\begin{aligned}&I_1^N\big (x^{k,N},y^{k,N}\big )\\&\quad =-\frac{\delta }{4}\left( \left| \left| (1-\delta ) x^{k,N}-\delta {\mathcal {C}}_N\nabla \varPsi ^N\big (x^{k,N}\big )+\sqrt{2\delta } {\mathcal {C}}_N^{1/2}\xi ^{k,N}\right| \right| _{{\mathcal {C}}_N}^2-\left| \left| x^{k,N}\right| \right| _{{\mathcal {C}}_N}^2\right) . \end{aligned}$$
  Expanding the above we obtain:
  6.16 $$\begin{aligned} I_1^N\big (x^{k,N},y^{k,N}\big )-\frac{\ell ^2\big (S^{k,N}-1\big )}{2}&= -\left( \frac{\delta ^2}{2}\left| \left| {\mathcal {C}}_N^{1/2}\xi ^{k,N}\right| \right| _{{\mathcal {C}}_N}^2 -\frac{\ell ^2}{2}\right) \nonumber \\&\quad +(r_{\varPsi }^N - r^N)+r_{\xi }^N+r_x^N, \end{aligned}$$
  where the difference $(r_{\varPsi }^N - r^N)$ is defined in (4.5) and we set
  6.17 $$\begin{aligned} r^N_{\xi }&:= -\frac{(\delta ^{3/2}-\delta ^{5/2})}{\sqrt{2}} \left\langle x^{k,N},{\mathcal {C}}_N^{1/2}\xi ^{k,N} \right\rangle _{{\mathcal {C}}_N}, \end{aligned}$$
  6.18 $$\begin{aligned} r^N_{x}&:= -\frac{\delta ^3}{4}\left| \left| x^{k,N}\right| \right| _{{\mathcal {C}}_N}^2. \end{aligned}$$
  For the reader’s convenience we rearrange (4.5) below:
  6.19 $$\begin{aligned} r_{\varPsi }^N - r^N&= \frac{\delta ^2-\delta ^3}{2}\left\langle x^{k,N},{\mathcal {C}}_N\nabla \varPsi ^N\big (x^{k,N}\big ) \right\rangle _{{\mathcal {C}}_N} \nonumber \\&\quad -\frac{\delta ^3}{4}\left| \left| {\mathcal {C}}_N\nabla \varPsi ^N\big (x^{k,N}\big )\right| \right| _{{\mathcal {C}}_N}^2 +\frac{\delta ^{5/2}}{\sqrt{2}}\left\langle {\mathcal {C}}_N\nabla \varPsi ^N\big (x^{k,N}\big ),{\mathcal {C}}_N^{1/2}\xi ^{k,N} \right\rangle _{{\mathcal {C}}_N}. \end{aligned}$$
  We come to bound all of the above terms, starting from (6.19). To this end, let us observe the following:
  6.20 $$\begin{aligned} \left| \left\langle x^{k,N},{\mathcal {C}}_N\nabla \varPsi ^N\big (x^{k,N}\big ) \right\rangle _{{\mathcal {C}}_N}\right| ^2&=\left| \sum _{i=1}^N x^{k,N}_i [\nabla \varPsi ^N\big (x^{k,N}\big )]_i\right| ^2 \end{aligned}$$
  6.21 $$\begin{aligned}&{\mathop {\le }\limits ^{(2.6)}} \left| \left| x^{k,N}\right| \right| _{s}^2 \Vert \nabla \varPsi ^N\big (x^{k,N}\big )\Vert _{-s}^2 {\mathop {\lesssim }\limits ^{(2.24)}} \left| \left| x^{k,N}\right| \right| _{s}^2. \end{aligned}$$
  Moreover,
  $$\begin{aligned} {\mathbb {E}}_k \left| \left| {\mathcal {C}}_N^{1/2} \xi ^{k,N}\right| \right| _{{\mathcal {C}}_N}^2 = {\mathbb {E}}_k \sum _{j=1}^N \left| \xi _j\right| ^2 = N, \end{aligned}$$
  hence
  $$\begin{aligned} \left| \left\langle {\mathcal {C}}_N\nabla \varPsi ^N\big (x^{k,N}\big ),{\mathcal {C}}_N^{1/2}\xi ^{k,N} \right\rangle _{{\mathcal {C}}_N}\right| ^2 \le \left| \left| {\mathcal {C}}_N\nabla \varPsi ^N\big (x^{k,N}\big )\right| \right| _{{\mathcal {C}}_N}^2\left| \left| {\mathcal {C}}_N^{1/2} \xi ^{k,N}\right| \right| _{{\mathcal {C}}_N}^2 {\mathop {\lesssim }\limits ^{(2.26)}}N. \end{aligned}$$
  From (6.19), (6.20), (2.26) and the above,
  6.22 $$\begin{aligned} {\mathbb {E}}_k \left| r_{\varPsi }^N-r^N\right| ^2 \lesssim \frac{\left| \left| x^{k,N}\right| \right| _{s}^2}{N^2}+\frac{1}{N^{3/2}}. \end{aligned}$$
  By (6.17),
  6.23 $$\begin{aligned} {\mathbb {E}}_k \left| r^N_{\xi }\right| ^2&\lesssim \frac{1}{N^{3/2}} {\mathbb {E}}_k\left| \left\langle x^{k,N},{\mathcal {C}}_N^{1/2}\xi ^{k,N} \right\rangle _{{\mathcal {C}}_N}\right| ^2\nonumber \\&= \frac{1}{N^{3/2}}{\mathbb {E}}_k \left( \sum _{i=1}^N \frac{x_i^{k,N} \xi _i^{k,N}}{\lambda _i} \right) ^2 = \frac{1}{\sqrt{N}}S^{k,N}, \end{aligned}$$
  where in the last equality we have used the fact that $\{\xi _i^{k,N}:i=1,\ldots ,N\}$ are independent, zero mean, unit variance normal random variables (independent of $x^{k,N}$) and (4.6). As for $r^N_{x}$,
  $$\begin{aligned} {\mathbb {E}}_k \left| r_x^N\right| ^2 \lesssim \frac{1}{N^3}\left| \left| x^{k,N}\right| \right| _{{\mathcal {C}}_N}^4{\mathop {=}\limits ^{(4.6)}}\frac{(S^{k,N})^2}{N}. \end{aligned}$$
  Lastly,
  $$\begin{aligned} {\tilde{r}}^N:=\frac{\delta ^2}{2}\left| \left| {\mathcal {C}}_N^{1/2}\xi ^{k,N}\right| \right| _{{\mathcal {C}}_N}^2 -\frac{\ell ^2}{2}=\frac{\ell ^2}{2}\left( \frac{1}{N}\sum _{j=1}^N\xi ^2_j-1\right) . \end{aligned}$$
  Since $\sum _{j=1}^N\xi ^2_j$ has chi-squared law, ${\mathbb {E}}_k\left| {\tilde{r}}^N\right| ^2\lesssim Var\left( N^{-1}\sum _{j=1}^N\xi ^2_j\right) \lesssim N^{-1}$, by (6.5). Combining all of the above, we obtain the desired bound.
- Proof of (6.13) From (6.10),
  $$\begin{aligned} I_2^N\big (x^{k,N},y^{k,N}\big )&=-\left[ \varPsi ^N(y^{k,N})-\varPsi ^N\big (x^{k,N}\big ) -\left\langle y^{k,N}-x^{k,N},\nabla \varPsi ^N\big (x^{k,N}\big ) \right\rangle \right] \\&\quad +\frac{1}{2}\left\langle y^{k,N}-x^{k,N},\nabla \varPsi ^N(y^{k,N})-\nabla \varPsi ^N\big (x^{k,N}\big ) \right\rangle \\&\quad +\frac{\delta }{2}\left( \left\langle x^{k,N},\nabla \varPsi ^N\big (x^{k,N}\big ) \right\rangle -\left\langle y^{k,N},\nabla \varPsi ^N(y^{k,N}) \right\rangle \right) =:\sum _{j=1}^3d_j, \end{aligned}$$
  where $d_j$ is the addend on line *j* of the above array. Using (2.22), (2.24), (2.6) and Lemma 6.1, we have
  $$\begin{aligned} {\mathbb {E}}_k \left| d_1\right| ^{2}\lesssim {\mathbb {E}}_k \left| \left| y^{k,N}-x^{k,N}\right| \right| _s^{2} \lesssim \frac{1+\left| \left| x^{k,N}\right| \right| _{s}^{2}}{\sqrt{N}}. \end{aligned}$$
  By the first inequality in (2.24),
  $$\begin{aligned} \left| \left| \nabla \varPsi ^N(y^{k,N})-\nabla \varPsi ^N\big (x^{k,N}\big )\right| \right| _{-s}\lesssim 1. \end{aligned}$$
  Consequently, again by (2.6) and Lemma 6.1,
  $$\begin{aligned} {\mathbb {E}}_k \left| d_2\right| ^{2}\lesssim {\mathbb {E}}_k \left| \left| y^{k,N}-x^{k,N}\right| \right| _s^{2}\lesssim \frac{1+\left| \left| x^{k,N}\right| \right| _{s}^{2}}{\sqrt{N}}. \end{aligned}$$
  Next, applying (2.6) and (2.24) gives
  $$\begin{aligned} \left| {d_3}\right|&\le \frac{\left| \left| x^{k,N}\right| \right| _{s}\left| \left| \nabla \varPsi ^N\big (x^{k,N}\big )\right| \right| _{-s} +\left| \left| y^{k,N}\right| \right| _{s}\left| \left| \nabla \varPsi ^N(y^{k,N})\right| \right| _{-s}}{\sqrt{N}}\\&\lesssim \frac{\left| \left| x^{k,N}\right| \right| _{s}+\left| \left| y^{k,N}\right| \right| _{s}}{\sqrt{N}} \lesssim \frac{\left| \left| x^{k,N}\right| \right| _{s}+\left| \left| y^{k,N}-x^{k,N}\right| \right| _{s}}{\sqrt{N}}. \end{aligned}$$
  Thus, applying Lemma 6.1 then gives the desired bound.
- Proof of (6.14) This follows directly from (2.25). $\square$

### Correlations between acceptance probability and noise $\xi ^{k,N}$

Recall the definition of $\gamma ^{k,N}$, Eq. (2.13), and let

6.24 $$\begin{aligned} \varepsilon ^{k,N}:= \gamma ^{k,N}{\mathcal {C}}_N^{1/2}\xi ^{k,N}. \end{aligned}$$

The study of the properties of $\varepsilon ^{k.N}$ is the object of the next two lemmata, which have a central role in the analysis: Lemma 6.5 (and Lemma 6.2) establishes the decay of correlations between the acceptance probability and the noise $\xi ^{k,N}$. Lemma 6.6 formalizes the heuristic arguments presented in Sect. 4.3.2.

#### Lemma 6.5

If Assumption 2.1 holds, then

6.25 $$\begin{aligned} \left| \left| {{\mathbb {E}}_k \varepsilon ^{k,N}}\right| \right| _{s}^2\lesssim \frac{1+\left| \left| x^{k,N}\right| \right| _{s}^2}{\sqrt{N}}. \end{aligned}$$

Therefore,

6.26 $$\begin{aligned} \left\langle {{\mathbb {E}}_k \varepsilon ^{k,N}},x^{k,N} \right\rangle _{s}{={\mathbb {E}}_k\left\langle \gamma ^{k,N}{\mathcal {C}}_N^{1/2}\xi ^{k,N},x^{k,N} \right\rangle _{s}}\lesssim \frac{1}{N^{1/4}}\left( 1+\left| \left| x^{k,N}\right| \right| _{s}^2\right) . \end{aligned}$$

#### Lemma 6.6

Let Assumption 2.1 hold. Then, with the notation introduced so far,

$$\begin{aligned} \left| {\mathbb {E}}_k \left| \left| \varepsilon ^{k,N}\right| \right| _{s}^2-{\mathrm{Trace}}_{{\mathcal {H}}^s}({\mathcal {C}}_s)\alpha _{\ell }\big (S^{k,N}\big )\right| \lesssim \frac{1+S^{k,N}+\left| \left| x^{k,N}\right| \right| _{s}}{N^{1/4}}. \end{aligned}$$

The proofs of the above lemmata can be found in Appendix B. Notice that if $\xi ^{k,N}$ and $\gamma ^{k,N}$ (equivalently $\xi ^{k,N}$ and $Q^{N}$) were uncorrelated, the statements of Lemmas 6.5 and 6.6 would be trivially true.

## Proof of Theorem 4.1

As explained in Sect. 5.1, due to the continuity of the map ${\mathcal {J}}_2$ (defined in Theorem 3.3), in order to prove Theorem 4.1 all we need to show is convergence in probability of ${\hat{w}}^N(t)$ to zero. Looking at the definition of ${\hat{w}}^N(t)$, Eq. (5.3), the convergence in probability (in $C([0,T];{\mathbb {R}})$) of ${\hat{w}}^N(t)$ to zero is consequence of Lemmas 7.1 and 7.2 below. We prove Lemma 7.1 in Sect. 7.1 and Lemma 7.2 in Sect. 7.2.

### Lemma 7.1

Let Assumption 2.1 hold and recall the definition (5.4) of the process $e^N(t)$; then

$$\begin{aligned} \lim _{N\rightarrow \infty }{\mathbb {E}}_{x^0}\left( \sup _{t\in [0,T]}\left| e^N(t)\right| \right) ^2=0. \end{aligned}$$

### Lemma 7.2

Let Assumption 2.1 hold and recall the definition (5.1) of the process $w^N(t)$; then

$$\begin{aligned} \lim _{N\rightarrow \infty }{\mathbb {E}}_{x^0}\left( \sup _{t\in [0,T]}\left| w^N(t)\right| \right) ^2=0. \end{aligned}$$

### Analysis of the drift

In view of what follows, it is convenient to introduce the piecewise constant interpolant of the chain $\{S^{k,N}\}_{k\in {\mathbb {N}}}$:

7.1 $$\begin{aligned} {\bar{S}}^{(N)}(t):=S^{k,N}, \quad t_k\le t<t_{k+1}, \end{aligned}$$

where $$t_k= k/\sqrt{N}$$.

#### Proof of Lemma 7.1

From (7.1), for any $t_k\le t<t_{k+1}$ we have

$$\begin{aligned} \int _0^tb_{\ell }({\bar{S}}^{(N)}_v)dv&= \int _{t_k}^tb_{\ell }({\bar{S}}^{(N)}_v)dv +\sum _{j=1}^{k-1}\int _{t_{j-1}}^{t_j}b_{\ell }({\bar{S}}^{(N)}_v)dv\\&=(t-t_k)b_{\ell }\big (S^{k,N}\big )+ \frac{1}{\sqrt{N}}\sum _{j=1}^{k-1}b_{\ell }(S^{j,N}). \end{aligned}$$

With this observation, we can then decompose $e^N(t)$ as

$$\begin{aligned} e^N(t)=e^N_1(t)- e^N_2(t), \end{aligned}$$

where

7.2 $$\begin{aligned} e^N_1(t)&:=(t-t_k) \big (b_{\ell }^{k,N}-b_{\ell }\big (S^{k,N}\big )\big )+\frac{1}{\sqrt{N}} \sum _{j=0}^{k-1}\left[ b_{\ell }^{j,N}-b_{\ell }(S^{j,N})\right] \end{aligned}$$

7.3 $$\begin{aligned} e_2^N(t)&:=\int _0^t \left[ b_{\ell }( S^{(N)}_v)-b_{\ell }({\bar{S}}^{(N)}_v) \right] dv. \end{aligned}$$

The result is now a consequence of Lemmas 7.3 and 7.4 below, which we first state and then consecutively prove. $\square$

#### Lemma 7.3

If Assumption 2.1 holds, then

$$\begin{aligned} \lim _{N\rightarrow \infty }{\mathbb {E}}_{x^0}\left( \sup _{t\in [0,T]} \left| e_1^N(t) \right| \right) ^2=0. \end{aligned}$$

#### Lemma 7.4

If Assumption 2.1 holds, then

$$\begin{aligned} \lim _{N\rightarrow \infty }{\mathbb {E}}_{x^0}\left( \sup _{t\in [0,T]} \left| e_2^N(t) \right| \right) ^2=0. \end{aligned}$$

#### Proof of Lemma 7.3

Denoting $E^{k,N}:=b_{\ell }^{k,N}-b_\ell \big (S^{k,N}\big )$, by (discrete) Jensen’s inequality we have

$$\begin{aligned} \sup _{t\in [0,T]}\left| e_1^N(t)\right| ^2&=\sup _{t\in [0,T]}\left| (t-t_k) E^{k,N}+\frac{1}{\sqrt{N}} \sum _{j=0}^{k-1}E^{k,N}\right| ^2\\&\lesssim \frac{1}{\sqrt{N}} \sum _{j=0}^{[T\sqrt{N}]-1}\left| E^{j,N}\right| ^2. \end{aligned}$$

Using Lemma 7.5 below, we obtain

$$\begin{aligned} \frac{1}{\sqrt{N}}\sum _{j=0}^{[T\sqrt{N}]-1}\left| E^{j,N}\right| ^2\lesssim \frac{1}{\sqrt{N}}\sum _{k=0}^{[T\sqrt{N}]-1}\frac{1+\big (S^{k,N}\big )^4 +\left| \left| x^{k,N}\right| \right| _{s}^4}{\sqrt{N}}. \end{aligned}$$

Taking expectations on both sides and applying Lemma 6.2 completes the proof. $\square$

#### Lemma 7.5

Let Assumption 2.1 hold. Then, for any $N \in {\mathbb {N}}$ and $k\in \{0, 1 ,\ldots , [T\sqrt{N}]\}$,

$$\begin{aligned} \left| E^{k,N} \right| ^2= \left| b_{\ell }^{k,N}-b_{\ell }\big (S^{k,N}\big )\right| ^2\lesssim \frac{1+\big (S^{k,N}\big )^4+\left| \left| x^{k,N}\right| \right| _{s}^4}{\sqrt{N}}. \end{aligned}$$

#### Proof of Lemma 7.5

Define

$$\begin{aligned} Y^N_k:=\frac{\left| \left| y^{k,N}\right| \right| _{{\mathcal {C}}_N}^2 -\left| \left| x^{k,N}\right| \right| _{{\mathcal {C}}_N}^2}{\sqrt{N}},\qquad {\tilde{Y}}^N_k:=2\ell (1-S^{k,N}). \end{aligned}$$

Then, from (4.19), (4.2), (1.12) and (1.14), we obtain

$$\begin{aligned} \left| b_{\ell }^{k,N}-b_{\ell }\big (S^{k,N}\big )\right| ^2=&\,\left| {\mathbb {E}}_k \left( \alpha ^N\big (x^{k,N},y^{k,N}\big )Y^N_k\right) - \alpha _\ell \big (S^{k,N}\big ){\tilde{Y}}^N_k\right| ^2 \\ \le&\, {\mathbb {E}}_k \left| \alpha ^N\big (x^{k,N},y^{k,N}\big )Y^N_k- \alpha _\ell \big (S^{k,N}\big ){\tilde{Y}}^N_k \right| ^2\\ \lesssim&\, \,{\mathbb {E}}_k \left[ \left| \alpha ^N\big (x^{k,N},y^{k,N}\big )\right| ^2\left| Y^N_k-{\tilde{Y}}^N_k \right| ^2\right] \\&+{\mathbb {E}}_k \left[ \left| {\tilde{Y}}^N_k \right| ^2\left| \alpha ^N\big (x^{k,N},y^{k,N}\big )- \alpha _\ell \big (S^{k,N}\big )\right| ^2\right] . \end{aligned}$$

Since $|\alpha ^N\big (x^{k,N},y^{k,N}\big )|\le 1$ and ${\tilde{Y}}^N_k$ is a function of $x^{k,N}$ only, we can further estimate the above as follows:

7.4 $$\begin{aligned} \left| b_{\ell }^{k,N}-b_{\ell }\big (S^{k,N}\big )\right| ^2\lesssim {\mathbb {E}}_k \left| Y^N_k-{\tilde{Y}}^N_k \right| ^2+\left| {\tilde{Y}}^N_k\right| ^2{\mathbb {E}}_k \left| \alpha ^N\big (x^{k,N},y^{k,N}\big )- \alpha _{\ell }\big (S^{k,N}\big )\right| ^2. \end{aligned}$$

From the definition of $I_1^N$, Eq. (6.9), we have

7.5 $$\begin{aligned} Y^N_k=-\frac{4}{\ell }I_1^N\big (x^{k,N},y^{k,N}\big ). \end{aligned}$$

Therefore,

$$\begin{aligned} Y^N_k-{\tilde{Y}}^N_k= -\frac{4}{\ell } \left[ I_1^N - \frac{\ell ^2}{2}\big (S^{k,N}-1\big ) \right] , \end{aligned}$$

which implies

$$\begin{aligned} {\mathbb {E}}_k (Y^N_k-{\tilde{Y}}^N_k)^2\lesssim&\,{\mathbb {E}}_k \left( I_1^N\big (x^{k,N},y^{k,N}\big )-\ell ^2 \big (S^{k,N}-1\big )/2\right) ^2\\ {\mathop {\lesssim }\limits ^{(6.12)}}&\frac{\left| \left| x^{k,N}\right| \right| _{s}^2}{N^2}+\frac{\big (S^{k,N}\big )^2}{\sqrt{N}}+\frac{1}{N}. \end{aligned}$$

As for the second addend in (7.4), Lemma 6.3 gives

$$\begin{aligned} \left| {\tilde{Y}}^N_k\right| ^2{\mathbb {E}}_k \left| \alpha ^N\big (x^{k,N},y^{k,N}\big )- \alpha _\ell (S^{k,N})\right| ^2\lesssim & {} (1+\big (S^{k,N}\big )^2)\left( \frac{1+\big (S^{k,N}\big )^2+\left| \left| x^{k,N}\right| \right| _{s}^2}{\sqrt{N}}\right) \\\lesssim & {} \frac{1+\big (S^{k,N}\big )^4+\left| \left| x^{k,N}\right| \right| _{s}^4}{\sqrt{N}}. \end{aligned}$$

Combining the above two bounds and (7.4) gives the desired result. $\square$

#### Proof of Lemma 7.4

By Jensen’s inequality,

$$\begin{aligned} \left( \sup _{t\in [0,T]}\left| \int _0^tb_{\ell }( S^{(N)}_v)-b_{\ell }({\bar{S}}^{(N)}_v)dv\right| \right) ^2\lesssim \int _0^T\left| b_{\ell }( S^{(N)}_v)-b_{\ell }({\bar{S}}^{(N)}_v)\right| ^2dv. \end{aligned}$$

Since $b_{\ell }$ is globally Lipschitz,

$$\begin{aligned} \int _0^T\left| b_{\ell }({\bar{S}}^N(v)) -b_{\ell }(S^N(v)) \right| ^2dv \lesssim&\,\int _0^T\left| {\bar{S}}^N(v) -S^N(v) \right| ^2dv\\ =&\,\sum _{k=0}^{[T\sqrt{N}]-1}\int _{t_k}^{t_{k+1}}\left| {\bar{S}}^N(v) -S^N(v)\right| ^2dv\\&+\int _{[T\sqrt{N}]}^{T}\left| {\bar{S}}^N(v) -S^N(v)\right| ^2dv\\ \lesssim&\,\frac{1}{\sqrt{N}}\sum _{k=0}^{[T\sqrt{N}]-1}(S^{k+1,N}-S^{k,N})^2. \end{aligned}$$

From (4.18) and (4.6),

$$\begin{aligned} \left| S^{k+1,N}-S^{k,N}\right|&\lesssim \frac{1}{N} \left( \Vert y^{k,N}\Vert _{{\mathcal {C}}^N}^2- \Vert x^{k,N}\Vert _{{\mathcal {C}}^N}^2 \right) \\&{\mathop {\lesssim }\limits ^{(7.5)}} \frac{1}{\sqrt{N}} I_1^N\big (x^{k,N},y^{k,N}\big )\\&= \frac{1}{\sqrt{N}} \left( I_1^N\big (x^{k,N},y^{k,N}\big ) - \frac{\ell ^2 \big (S^{k,N}-1\big )}{2} \right) + \frac{1}{\sqrt{N}}\frac{\ell ^2 \big (S^{k,N}-1\big )}{2}. \end{aligned}$$

Combining the above with (6.12) we obtain

7.6 $$\begin{aligned} {\mathbb {E}}_k {(S^{k+1,N}-S^{k,N})^2} \lesssim \frac{1+\big (S^{k,N}\big )^2+\left| \left| x^{k,N}\right| \right| _{s}^2}{N}. \end{aligned}$$

Taking expectations and applying Lemma 6.2 concludes the proof. $\square$

### Analysis of noise

#### Proof of Lemma 7.2

Notice that we can write $w^N$ as the linear interpolation

$$\begin{aligned} w^N(t)=(N^{1/2}t-k)M^{k,N}+(k+1-N^{1/2}t)M^{k-1,N}\qquad \forall t_k\le t<t_{k+1}, \end{aligned}$$

of the array

$$\begin{aligned} M^{k,N}:=\frac{1}{N^{1/4}}\sum _{j=0}^{k-1}D^{j,N},\qquad \forall k=1,\ldots ,[T\sqrt{N}]+1. \end{aligned}$$

It follows from the definition of $D^{k,N}$ in (4.17) and Lemma 6.2 that $\{M^{k,N}\}_{k\ge 1}$ is a discrete-time ${\mathbb {P}}_{x^0}$-martingale with respect to the filtration generated by $\{x^{k,N}\}_{k\ge 1}$. Since,

$$\begin{aligned} \sup _{t\in [0,T]}\left| w^N(t)\right| =\sup _{k\in \{1,\ldots ,[T\sqrt{N}]+1\}}\left| M^{k,N}\right| , \end{aligned}$$

Doob’s $L^p$ inequality implies that

$$\begin{aligned} {\mathbb {E}}_{x^0}\left( \sup _{t\in [0,T]}\left| w^N(t)\right| \right) ^2&\lesssim {\mathbb {E}}_{x^0}\left( \sup _{k\in \{1,\ldots ,[T\sqrt{N}]+1\}}\left| M^{k,N}\right| ^2 \right) \\&=\frac{1}{\sqrt{N}}\sum _{k=0}^{[T\sqrt{N}]}{\mathbb {E}}_{x^0}\left| {D^{k,N}}\right| ^2, \end{aligned}$$

where the equality follows from the independence of the increments of $\{M^{k,N}\}_{k\ge 1}$. From the definition of $D^{k,N}$, Eq. (4.17), we have that

$$\begin{aligned} \frac{{\mathbb {E}}_{x^0}\left| D^{k,N}\right| ^2}{\sqrt{N}}&={\mathbb {E}}_{x^0}\left[ S^{k+1,N}-S^{k,N} -{\mathbb {E}}_k \left( {S^{k+1,N}-S^{k,N}}\right) \right] ^2\\&\lesssim {\mathbb {E}}_{x^0}\left| S^{k+1,N}-S^{k,N} \right| ^2{\lesssim }\frac{1}{{N}}, \end{aligned}$$

where the last inequality is a consequence of (7.6) and Lemma 6.2. The result follows immediately. $\square$

## Proof of Theorem 4.2

The idea behind the proof is the same as in the previous Sect. 7. First we introduce the piecewise constant interpolant of the chain $\{x^{k,N}\}_{k\in {\mathbb {N}}}$

8.1 $$\begin{aligned} {\bar{x}}^{(N)}(t)=x^{k,N} \quad \text{ for } \,\, t_k\le t < t_{k+1}. \end{aligned}$$

Due to the continuity of the map ${\mathcal {J}}_1$ (Theorem 3.3), all we need to prove is the weak convergence of ${\hat{\eta }}^N(t)$ to zero (see Sect. 5.2). Looking at the definition of ${\hat{\eta }}^N(t)$, Eq. (5.6), this follows from Lemmas 8.1, 8.2 and 8.3 below. We prove Lemmas 8.1 and 8.2 in Sect. 8.1 and Lemma 8.3 in Sect. 8.2.

### Lemma 8.1

Let Assumption 2.1 hold and recall the definition (5.8) of the process $d^N(t)$; then

$$\begin{aligned} \lim _{N\rightarrow \infty }{\mathbb {E}}_{x^0}\left( \sup _{t\in [0,T]}\left| d^N(t)\right| \right) ^2=0. \end{aligned}$$

### Lemma 8.2

If Assumption 2.1 holds, then $\upsilon ^N$ (defined in (5.9)) converges in probability in $C([0,T]; {\mathcal {H}}^s)$ to zero.

### Lemma 8.3

Let Assumption 2.1 hold. Then the interpolated martingale difference array $\mathfrak {\eta }^N(t)$ defined in (5.7) converges weakly in $C([0,T]; {\mathcal {H}}^s)$ to the stochastic integral $\eta (t)$, defined in Eq. (5.11).

### Analysis of drift

#### Proof (Lemma 8.1)

For all $t\in [t_k,t_{k+1})$, we can write

$$\begin{aligned} (t-t_k) \varTheta (x^{k,N},S^{k,N})+\frac{1}{\sqrt{N}}\sum _{j=0}^{k-1}\varTheta \big (x^{j,N},S^{j,N}\big ) =\int _0^t\varTheta \big ({\bar{x}}^{(N)}(v),{\bar{S}}^{(N)}(v)\big )dv. \end{aligned}$$

Therefore, we can decompose $d^N(t)$ as

$$\begin{aligned} d^N(t)=d^N_1(t)+d^N_2(t), \end{aligned}$$

where

$$\begin{aligned} d_1^N(t):=(t-t_k)\left[ \varTheta ^{k,N}-\varTheta \big (x^{k,N},S^{k,N}\big )\right] +\frac{1}{\sqrt{N}}\sum _{j=0}^{k-1}\left[ \varTheta ^{j,N}-\varTheta \big (x^{j,N},S^{j,N}\big )\right] \end{aligned}$$

and

$$\begin{aligned} d_2^N(t):=\int _0^t \left[ \varTheta \big ({\bar{x}}^N(v), {\bar{S}}^N (v)\big ) - \varTheta \big ({x}^{(N)}(v), {S}^{(N)}(v)\big )\right] dv. \end{aligned}$$

The statement is now a consequence of Lemmas 8.4 and 8.5. $\square$

#### Lemma 8.4

If Assumption 2.1 holds, then

$$\begin{aligned} \lim _{N\rightarrow \infty }{\mathbb {E}}_{x^0}\left( \sup _{t\in [0,T]}\left| \left| d_1^N(t)\right| \right| _{s} \right) ^2= 0. \end{aligned}$$

#### Lemma 8.5

If Assumption 2.1 holds, then

$$\begin{aligned} \lim _{N\rightarrow \infty }{\mathbb {E}}_{x^0}\left( \sup _{t\in [0,T]}\left| \left| d_2^N(t)\right| \right| _{s} \right) ^2= 0. \end{aligned}$$

Before proving Lemma 8.4, we state and prove the following Lemma 8.6. We then consecutively prove Lemmas 8.4, 8.5 and 8.2. Recall the definitions of $\varTheta$ and $\varTheta ^{k,N}$, equations (4.23) and (4.21), respectively.

#### Lemma 8.6

Let Assumption 2.1 hold and set

8.2 $$\begin{aligned} p^{k,N}:=\varTheta ^{k,N}-\varTheta (x^{k,N},S^{k,N}). \end{aligned}$$

Then

$$\begin{aligned} {\mathbb {E}}_{x^0}\left| \left| p^{k,N}\right| \right| _{s}^2\lesssim&\sum _{j=N+1}^\infty (\lambda _jj^s)^4+\frac{1}{\sqrt{N}}. \end{aligned}$$

#### Proof of Lemma 8.6

Recalling (4.26) and (6.24), we have

8.3 $$\begin{aligned} \left| \left| p^{k,N}\right| \right| _{s}^2&\lesssim \sqrt{N}\left| \left| {\mathbb {E}}_k\varepsilon ^N_k\big (x^{k,N}\big )\right| \right| _{s}^2 \end{aligned}$$

8.4 $$\begin{aligned}&\quad + \left| \left| \alpha _\ell \big (S^{k,N}\big )F\big (x^{k,N}\big )- \left[ {\mathbb {E}}_k \alpha ^N\big (x^{k,N},y^{k,N}\big )\right] \big (x^{k,N}+{\mathcal {C}}_N\nabla \varPsi ^N\big (x^{k,N}\big )\big )\right| \right| _{s}^2, \end{aligned}$$

where the function *F* that appears in the above has been defined in Lemma 2.1. The term on the RHS of (8.3) has been studied in Lemma 6.5. To estimate the addend in (8.4) we use (2.25), the boundedness of $\alpha _{\ell }$ and Lemma 6.3. A straightforward calculation then gives

$$\begin{aligned} (8.4)\lesssim&\, \left[ \alpha _\ell \big (S^{k,N}\big ) - {\mathbb {E}}_k \alpha ^N\big (x^{k,N},y^{k,N}\big )\right] ^2\left| \left| x^{k,N}+{\mathcal {C}}_N\nabla \varPsi ^N\big (x^{k,N}\big )\right| \right| _{s}^2\\&+ \left| \left| \alpha _{\ell }\big (S^{k,N}\big ) \left[ F\big (x^{k,N}\big ) - \big (x^{k,N}+{\mathcal {C}}_N\nabla \varPsi ^N\big (x^{k,N}\big )\big ) \right] \right| \right| _{s}^2\\ \lesssim&\, \frac{1+\big (S^{k,N}\big )^4+\left| \left| x^{k,N}\right| \right| _{s}^4}{\sqrt{N}} +\left| \left| {\mathcal {C}}\nabla \varPsi \big (x^{k,N}\big )-{\mathcal {C}}_N\nabla \varPsi ^N\big (x^{k,N}\big )\right| \right| _{s}^2. \end{aligned}$$

From the definition of $\varPsi ^N$ and $\nabla \varPsi ^N$, Eqs. (1.5) and (2.23), respectively,

$$\begin{aligned} \left| \left| {\mathcal {C}}\nabla \varPsi \big (x^{k,N}\big )-{\mathcal {C}}_N\nabla \varPsi ^N\big (x^{k,N}\big )\right| \right| _{s}^2&= \left| \left| {\mathcal {C}}\nabla \varPsi \big (x^{k,N}\big )-{\mathcal {C}}_ N {\mathcal {P}}^N\big (\nabla \varPsi \big (x^{k,N}\big )\big )\right| \right| _{s}^2 \\&=\sum _{j=N+1}^\infty (\lambda _jj^s)^4{\mathbb {E}}\left[ j^{-2s}\big (\nabla \varPsi \big (x^{k,N}\big )\big )_j^2 \right] \\&\lesssim \sum _{j=N+1}^\infty (\lambda _jj^s)^4, \end{aligned}$$

having used (2.24) in the last inequality. The statement is now a consequence of Lemma 6.2. $\square$

#### Proof of Lemma 8.4

Following the analogous steps to those taken in the proof of Lemma 7.3, the proof is a direct consequence of Lemma 8.6, after observing that the summation $\sum _{j=N+1}^\infty (\lambda _jj^s)^4$ is the tail of a convergent series hence it tends to zero as $N \rightarrow \infty$. $\square$

#### Proof of Lemma 8.5

By the definition of $\varTheta$, Eq. (4.23), we have

$$\begin{aligned}&\left| \left| \varTheta ({\bar{x}}^N(t), {\bar{S}}^N (t)) - \varTheta ({x}^N(t), {S}^N (t)) \right| \right| _{s}\\&\quad =\left| \left| F({\bar{x}}^N(t))h_{\ell }({\bar{S}}^N(t)) - F({x}^{(N)}(t))h_{\ell }({S}^{(N)}(t)) \right| \right| _{s}. \end{aligned}$$

Applying (2.20) and (2.25) and using the fact $h_\ell$ is globally Lipschitz and bounded, we get

$$\begin{aligned}&\left| \left| \varTheta ({\bar{x}}^N(t), {\bar{S}}^N (t)) - \varTheta ({x}^N(t), {S}^N (t)) \right| \right| _{s}\\&\quad \lesssim \left| \left| {\bar{x}}^N(t)-{x}^{(N)}(t)\right| \right| _{s}+(1+\left| \left| {\bar{x}}^N(t)\right| \right| _{s})\left| {\bar{S}}^N (t)-S^{(N)} (t)\right| . \end{aligned}$$

Thus, from the definitions (1.16), (7.1), (1.9) and (8.1), if $t_k\le t<t_{k+1}$, we have

$$\begin{aligned}&\left| \left| \varTheta ({\bar{x}}^N(t), {\bar{S}}^N (t)) - \varTheta ({x}^N(t), {S}^N (t)) \right| \right| _{s}\\&\quad \lesssim (t-k\sqrt{N})\left| \left| x^{k+1,N}-x^{k,N}\right| \right| _{s}\\&\qquad +(t-k\sqrt{N})\left( 1+\left| \left| x^{k,N}\right| \right| _{s}\right) \left| S^{k+1,N}-S^{k,N}\right| . \end{aligned}$$

Applying (6.3) and (7.6) one then concludes

$$\begin{aligned}&{\mathbb {E}}_k \left| \left| \varTheta ({\bar{x}}^N(t), {\bar{S}}^N (t)) - \varTheta ({x}^N(t), {S}^N (t)) \right| \right| _{s}^2\\&\quad \lesssim (t-k\sqrt{N})^2\left( \frac{1+\left| \left| x^{k,N}\right| \right| _{s}^2}{\sqrt{N}} +\frac{\left| \left| x^{k,N}\right| \right| _{s}^4+\big (S^{k,N}\big )^4}{N}\right) \end{aligned}$$

The remainder of the proof is analogous to the proof of Lemma 7.4. $\square$

#### Proof of Lemma 8.2

For any arbitrary but fixed $\varepsilon >0$, we need to argue that

$$\begin{aligned} \lim _{N\rightarrow \infty }{\mathbb {P}}\left[ \sup _{t\in [0,T]} \left| \left| \upsilon ^N(t)\right| \right| _{s}\ge \varepsilon \right] =0. \end{aligned}$$

From the definition of $\upsilon ^N$ we have

$$\begin{aligned} \sup _{t\in [0,T]}\left| \left| \upsilon ^N(t)\right| \right| _{s}\le \int _0^T \left| \left| F(x^{(N)}(v))\right| \right| _{s}\left| S^{(N)}(v)-S(v)\right| dv. \end{aligned}$$

Using (2.21) and the fact that $\left| \left| x^{(N)}(t)\right| \right| _{s}\le \left| \left| x^{k,N}\right| \right| _{s}+\left| \left| x^{k+1,N}\right| \right| _{s}$ (which is a simple consequence of (1.9)), for any $t\in [t_k,t_{k+1})$

$$\begin{aligned} \sup _{t\in [0,T]}\left| \left| \upsilon ^N(t)\right| \right| _{s}&\le \left( \sup _{t\in [0,T]} \left| S^{(N)}(t)-S(t)\right| \right) \int _0^T\left| \left| F(x^{(N)}(v))\right| \right| _{s}dv\\&\lesssim \underbrace{\left( \sup _{t\in [0,T]}\left| S^{(N)}(t)-S(t)\right| \right) }_{=:a^N}\underbrace{\left( 1+\frac{1}{\sqrt{N}} \sum _{j=0}^{[T\sqrt{N}]-1}\left| \left| x^{j,N}\right| \right| _{s}\right) }_{=:u^N}. \end{aligned}$$

Using Markov’s inequality and Lemma 6.2, given any $\delta >0$, it is straightforward to find constant *M* such that ${\mathbb {P}}\left[ u^N> M\right] \le \delta$ for every $N\in {\mathbb {N}}$. Thus

$$\begin{aligned} {\mathbb {P}}\left[ \sup _{t\in [0,T]}\left| \left| \upsilon ^N(t)\right| \right| _{s}\ge \varepsilon \right]&\le {\mathbb {P}}\left[ a^N u^N\ge \varepsilon \right] \\&= {\mathbb {P}}[a^N u^N\ge \varepsilon , u^N\le M]+ {\mathbb {P}}[a^N u^N\ge \varepsilon , u^N> M]\\&\le {\mathbb {P}}\left[ a^N\ge \varepsilon /M\right] +{\mathbb {P}}\left[ u^N> M\right] \le {\mathbb {P}}\left[ a^N\ge \varepsilon /M\right] +\delta . \end{aligned}$$

Given that the $\delta$ was arbitrary, the result then follows from the fact that $S^{(N)}$ converges in probability to *S* (Theorem 4.1). $\square$

### Analysis of noise

The proof of Lemma 8.3 is based on [14, Lemma 8.9]. For the reader’s convenience, we restate [14, Lemma 8.9] below as Lemma 8.7. In order to state such a lemma let us introduce the following notation and definitions. Let $k_N:[0,T] \rightarrow {\mathbb {Z}}_+$ be a sequence of nondecreasing, right continuous functions indexed by *N*, with $k_N(0)=0$ and $k_N(T)\ge 1$. Let ${\mathcal {H}}$ be any Hilbert space and $\{X^{k,N}, {\mathcal {F}}^{k,N}\}_{0\le k \le k_N(T)}$ be a ${\mathcal {H}}$-valued martingale difference array (MDA), i.e. a double sequence of random variables such that ${\mathbb {E}}[X^{k,N}\vert {\mathcal {F}}_{k-1}^N ]=0$, ${\mathbb {E}}[\Vert { X^{k,N}}\Vert ^2\vert {\mathcal {F}}_{k-1}^N ]< \infty$ almost surely and sigma-algebras ${\mathcal {F}}^{k-1, N} \subseteq {\mathcal {F}}^{k,N}$. Consider the process ${\mathcal {X}}^N(t)$ defined by

$$\begin{aligned} {\mathcal {X}}^N(t):=\sum _{k=1}^{k_N(t)}X^{k,N}, \end{aligned}$$

if $k_N(t)\ge 1$ and $k_N(t) > \lim _{v\rightarrow 0+} k_N(t-v)$ and by linear interpolation otherwise. With this set up we recall the following result.

#### Lemma 8.7

**(Lemma 8.9** [14]**)** Let $D:{\mathcal {H}}\rightarrow {\mathcal {H}}$ be a self-adjoint positive definite trace class operator on $({\mathcal {H}}, \left| \left| \cdot \right| \right| )$. Suppose the following limits hold in probability

(i) there exists a continuous and positive function $f:[0,T]\rightarrow {\mathbb {R}}_+$ such that
  $$\begin{aligned} \lim _{N\rightarrow \infty } \sum _{k=1}^{k_N(T)} {\mathbb {E}}\bigg ({\left| \left| X^{k,N}\right| \right| }^2\vert {\mathcal {F}}_{k-1}^N\bigg )= {\mathrm{Trace}}_{{\mathcal {H}}}(D) \int _0^T f(t) dt \, ; \end{aligned}$$
(ii) if $\{{\phi }_j\}_{j\in {\mathbb {N}}}$ is an orthonormal basis of ${\mathcal {H}}$ then
  $$\begin{aligned} \lim _{N\rightarrow \infty } \sum _{k=1}^{k_N(T)} {\mathbb {E}}\bigg (\langle X^{k,N},{\phi }_j \rangle \langle X^{k,N},{\phi }_i \rangle \vert {\mathcal {F}}_{k-1}^N\bigg )=0\, \quad \text{ for } \text{ all } \,\, i\ne j\, ; \end{aligned}$$
(iii) for every fixed $\epsilon >0$,
  $$\begin{aligned} \lim _{N \rightarrow \infty } \sum _{k=1}^{k_N(T)} {\mathbb {E}}\bigg ({\left| \left| X^{k,N}\right| \right| }^2 \mathbf{1}_{\left\{ {\left| \left| X^{k,N}\right| \right| }^2\ge \epsilon \right\} } \vert {\mathcal {F}}_{k-1}^N \bigg )=0, \qquad \text{ in } \text{ probability }, \end{aligned}$$

where ${\mathbf {1}}_A$ denotes the indicator function of the set *A*. Then the sequence ${\mathcal {X}}^N$ converges weakly in $C([0,T]; {\mathcal {H}}^s)$ to the stochastic integral $t\mapsto \int _0^t \sqrt{f(v)} dW_v$, where $W_t$ is a ${\mathcal {H}}$-valued *D*-Brownian motion.

#### Proof of Lemma 8.3

We apply Lemma 8.7 in the Hilbert space ${\mathcal {H}}^s$, with $k_N(t)=[t\sqrt{N}]$, $X^{k,N}=L^{k,N}/{N}^{1/4}$ [$L^{k,N}$ is defined in (4.22)] and ${\mathcal {F}}_k^N$ the sigma-algebra generated by $\{\gamma ^{h,N}, \xi ^{h,N}, \, 0\le h\le k\}$ to study the sequence $\eta ^N(t)$, defined in (5.7). We now check that the three conditions of Lemma 8.7 hold in the present case.

(i) Note that by the definition of $L^{k,N}$, ${\mathbb {E}}[L^{k,N}\vert {\mathcal {F}}_{k-1}^N]={\mathbb {E}}_k [L^{k,N}]$ almost surely. We need to show that the limit
  8.5 $$\begin{aligned} \lim _{N\rightarrow \infty }\frac{1}{\sqrt{N}}\sum _{k=0}^{[T\sqrt{N}]} {\mathbb {E}}_k \left| \left| L^{k,N}\right| \right| _{s}^2 = 2 \, {\mathrm{Trace}}_{{\mathcal {H}}^s}({\mathcal {C}}_s) \int _0^T h_{\ell }(S(u))du, \end{aligned}$$
  holds in probability. By (4.28),
  $$\begin{aligned} \frac{1}{\sqrt{N}} {\mathbb {E}}_k \left| \left| L^{k,N}\right| \right| _{s}^2&= {\mathbb {E}}_k \left| \left| x^{k+1,N}-x^{k,N}\right| \right| _{s}^2 - \left| \left| {\mathbb {E}}_k\left( x^{k+1,N}-x^{k,N}\right) \right| \right| _{s}^2. \end{aligned}$$
  From the above, if we prove
  8.6 $$\begin{aligned} {\mathbb {E}}_{x^0}\sum _{k=0}^{[T\sqrt{N}]}\left| \left| {\mathbb {E}}_k\left( x^{k+1,N}-x^{k,N}\right) \right| \right| _{s}^2 \rightarrow 0 \quad \text{ as } N\rightarrow \infty , \end{aligned}$$
  and that
  8.7 $$\begin{aligned}&\lim _{N\rightarrow \infty }\sum _{k=0}^{[T\sqrt{N}]} {\mathbb {E}}_k \left| \left| x^{k+1,N}-x^{k,N}\right| \right| _{s}^2 \nonumber \\&\quad = 2 \, {\mathrm{Trace}}_{{\mathcal {H}}^s}({\mathcal {C}}_s) \int _0^T h_{\ell }(S(u))du, \quad \text{ in } \text{ probability }, \end{aligned}$$
  then (8.5) follows. We start by proving (8.6):
  $$\begin{aligned} \left| \left| {\mathbb {E}}_k\left( x^{k+1,N}-x^{k,N}\right) \right| \right| _{s}^2 {\mathop {\lesssim }\limits ^{(2.14)}}&\frac{1}{N}\left| \left| x^{k,N}+{\mathcal {C}}_N \nabla \varPsi ^N(x^{k,N})\right| \right| _{s}^2 \\&+\frac{1}{\sqrt{N}} \left| \left| {\mathbb {E}}_k \left( \gamma ^{k,N}({\mathcal {C}}_N)^{1/2}\xi ^{k,N}\right) \right| \right| _{s}^2\\ \lesssim&\, \frac{1}{N} \left( 1+ \left| \left| x^{k,N}\right| \right| _{s}^2\right) , \end{aligned}$$
  where the last inequality follows from (2.25) and (6.25). The above and (6.7) prove (8.6). We now come to (8.7):
  $$\begin{aligned}&\left| \sum _{k=0}^{[T\sqrt{N}]} {\mathbb {E}}_k \left| \left| x^{k+1,N}-x^{k,N}\right| \right| _{s}^2-2 \, {\mathrm{Trace}}_{{\mathcal {H}}^s}({\mathcal {C}}_s) \int _0^T h_{\ell }(S(u))du \right| \\&\quad {\mathop {\lesssim }\limits ^{(2.14)}} \frac{1}{N}\sum _{k=0}^{[T\sqrt{N}]} {\mathbb {E}}_k \left| \left| x^{k,N}+{\mathcal {C}}_N \nabla \varPsi ^N(x^{k,N})\right| \right| _{s}^2\\&\quad \qquad + \frac{1}{N^{3/4}}\sum _{k=0}^{[T\sqrt{N}]} {\mathbb {E}}_k \left| \langle x^{k,N}+{\mathcal {C}}_N \nabla \varPsi ^N(x^{k,N}), {\mathcal {C}}_N^{1/2}\xi ^{k,N}\rangle _s\right| \\&\quad \qquad + \left| \frac{2\ell }{\sqrt{N}}\sum _{k=0}^{[T\sqrt{N}]}{\mathbb {E}}_k \left| \left| \gamma ^{k,N}{\mathcal {C}}_N^{1/2}\xi ^{k,N}\right| \right| _{s}^2 -2 \, {\mathrm{Trace}}_{{\mathcal {H}}^s}({\mathcal {C}}_s) \int _0^T h_{\ell }(S(u))du \right| .\\ \end{aligned}$$
  The first two addends tend to zero in $L_1$ as *N* tends to infinity due to (2.25), (2.27) and Lemma 6.2. As for the third addend, we decompose it as follows
  8.8 $$\begin{aligned}&\left| \frac{2\ell }{\sqrt{N}}\sum _{k=0}^{[T\sqrt{N}]}{\mathbb {E}}_k \left| \left| \gamma ^{k,N}{\mathcal {C}}_N^{1/2}\xi ^{k,N}\right| \right| _{s}^2 -2 \, {\mathrm{Trace}}_{{\mathcal {H}}^s}({\mathcal {C}}_s) \int _0^T h_{\ell }(S(u))du \right| \nonumber \\&\quad {\mathop {\lesssim }\limits ^{(1.13), (6.24)}} \left| \frac{\ell }{\sqrt{N}}\sum _{k=0}^{[T\sqrt{N}]}{\mathbb {E}}_k \left| \left| \varepsilon ^{k,N}\right| \right| _{s}^2 - \frac{\ell }{\sqrt{N}}\sum _{k=0}^{[T\sqrt{N}]}{\mathrm{Trace}}_{{\mathcal {H}}^s}({\mathcal {C}}_s)\alpha _{\ell }\big (S^{k,N}\big )\right| \nonumber \\&\qquad \qquad + \left| \frac{1}{\sqrt{N}}\sum _{k=0}^{[T\sqrt{N}]}{\mathrm{Trace}}_{{\mathcal {H}}^s}({\mathcal {C}}_s)h_{\ell }\big (S^{k,N}\big )- {\mathrm{Trace}}_{{\mathcal {H}}^s}({\mathcal {C}}_s) \int _0^T h_{\ell }(S(u))du\right| . \end{aligned}$$
  Convergence to zero in $L^1$ of the first term in the above follows from Lemmas 6.2 and 6.6. As for the term in (8.8), we use the identity
  $$\begin{aligned} \int _0^Th_{\ell }({\bar{S}}^{(N)}(u))du =\left( T-\frac{[T\sqrt{N}]}{\sqrt{N}}\right) h_{\ell }\big (S^{[T\sqrt{N}],N}\big ) +\frac{1}{\sqrt{N}}\sum _{k=0}^{[T\sqrt{N}]}h_{\ell }\big (S^{k,N}\big ), \end{aligned}$$
  to further split it, obtaining:
  8.9 $$\begin{aligned} (8.8)\lesssim&\, \left| \int _0^T h_{\ell }({\bar{S}}^{(N)}(u))- h_{\ell }(S^{(N)}(u))du \right| \end{aligned}$$
  8.10 $$\begin{aligned}&+\left| \int _0^T h_{\ell }(S^{(N)}(u))- h_{\ell }(S(u))du \right| \end{aligned}$$
  8.11 $$\begin{aligned}&+\left( T-\frac{[T\sqrt{N}]}{\sqrt{N}}\right) h_{\ell }(S^{[T\sqrt{N}],N}). \end{aligned}$$
  Convergence (in $L_1$) of (8.9) to zero follows with the same calculations leading to (7.6), the global Lipschitz property of $h_{\ell }$, and Lemma 6.2. The addend in (8.10) tends to zero in probability since $S^{(N)}$ tends to *S* in probability in $C([0,T];{\mathbb {R}})$ (Theorem 4.1) and the third addend is clearly small. The limit (8.7) then follows.
(ii) Condition (ii) of Lemma 8.7 can be shown to hold with similar calculations, so we will not show the details.
(iii) Using (6.3), the last bound follows a calculation completely analogous to the one in [14, Section 8.2]. We omit the details here. $\square$

## Appendix: Proofs of the results in Sect. 2

### Proof of Lemma 2.1

The bounds (2.20) are a consequence of (2.19). We show how to obtain the second bound in (2.20):

$$\begin{aligned} \left| \left| {\mathcal {C}}\nabla \varPsi (x)-{\mathcal {C}}\nabla \varPsi (y)\right| \right| _{s}^2&= \sum _{j=1}^{\infty } \lambda _j^4 j^{2s} \left[ \left( \nabla \varPsi (x)-\nabla \varPsi (y)\right) _j\right] ^2\\&=\sum _{j=1}^{\infty } (\lambda _j j^{s})^4 j^{-2s} \left[ \left( \nabla \varPsi (x)-\nabla \varPsi (y)\right) _j\right] ^2\\&\lesssim \Vert \nabla \varPsi (x)-\nabla \varPsi (y)\Vert _{-s}^2 {\mathop {\lesssim }\limits ^{(2.19)}} \Vert x-y\Vert _s^2, \end{aligned}$$

where in the above we have used (2.17) and $\left( \nabla \varPsi (x)-\nabla \varPsi (y)\right) _j$ denotes the *j*th component of the vector $\nabla \varPsi (x)-\nabla \varPsi (y)$. With analogous calculations one can obtain the first bound in (2.20). As for the second equation in (2.21):

$$\begin{aligned} \left| \left| F(z)\right| \right| _{s}&\lesssim \left| \left| z\right| \right| _{s}+ \Vert {\mathcal {C}}\nabla \varPsi (z)\Vert _{s} {\mathop {\lesssim }\limits ^{(2.20)}} 1+ \left| \left| z\right| \right| _{s}. \end{aligned}$$

Similarly for the first bound in (2.21). The proof of Eq. (2.22) is standard, so we only sketch it: consider a line joining points *x* and *y*, $\gamma (t)= x+t(y-x), t \in [0,1]$. Then

$$\begin{aligned} \varPsi (\gamma (1))-\varPsi (\gamma (0))&=\varPsi (y)-\varPsi (x)\\&= \int _0^1 dt \,\left\langle \nabla \varPsi (\gamma (t)), y-x \right\rangle \lesssim \left| \left| y-x\right| \right| _{s}, \end{aligned}$$

having used (2.19) and (2.6) in the last inequality. An analogous calculation to the above can be done for $\varPsi ^N$, after proving (2.24) below. $\square$

### Proof of Lemma 2.2

The bounds (2.24) and (2.25) are just consequences of the definition of $\varPsi ^N$ and $\nabla \varPsi ^N$ and the analogous properties of $\varPsi$. For the sake of clarity we just spell out how to obtain (2.25):

$$\begin{aligned} \left| \left| {\mathcal {C}}_N \nabla \varPsi ^N(x)\right| \right| _{s}^2&{\mathop {=}\limits ^{(2.23)}} \left| \left| {\mathcal {C}}_N {\mathcal {P}}^N\nabla \varPsi ({\mathcal {P}}^N(x))\right| \right| _{s}^2 = \sum _{j=1}^N j^{2s}\lambda _j^4\left[ \nabla \varPsi ({\mathcal {P}}^N(x)) \right] _j^2\\&\le \sum _{j=1}^{\infty } j^{2s}\lambda _j^4\left[ \nabla \varPsi ({\mathcal {P}}^N(x)) \right] _j^2\le \left| \left| {\mathcal {C}}\nabla \varPsi ({\mathcal {P}}^N(x))\right| \right| _{s}^2{\mathop {\lesssim }\limits ^{(2.20)}}1. \end{aligned}$$

As for (2.26), using (2.17):

$$\begin{aligned} \Vert {\mathcal {C}}_N \nabla \varPsi ^N(x)\Vert _{{\mathcal {C}}_N}^2&=\sum _{j=1}^{N} \lambda _j^2 \left[ \left( \nabla \varPsi ^N(x)\right) _j\right] ^2 \lesssim \sum _{j=1}^{\infty } j^{-2s} \left[ \left( \nabla \varPsi ^N(x)\right) _j\right] ^2 \\&= \Vert \nabla \varPsi ^N(x)\Vert _{-s}^2\lesssim 1. \end{aligned}$$

$$\square $$

## Appendix: Proofs of Lemmas 6.5 and 6.6

To prove Lemmas 6.5 and 6.6 we decompose $Q^N(x^{k,N}, \xi ^{k,N})$ into the sum of a term that depends on $\xi _j^{k,N}$ (the *j*th component of $\xi ^{k,N})$, $Q^N_j$ and one that is independent of $\xi _j$, $Q_{j,\perp }^N$:

$$\begin{aligned} Q^N=Q^N_j+Q_{j,\perp }^N, \end{aligned}$$

where

B.1 $$\begin{aligned} Q^N_j&:=\left( \frac{\ell ^{5/2}}{\sqrt{2}N^{5/4}} -\frac{\ell ^{3/2}}{\sqrt{2}N^{3/4}}\right) \frac{x^{k,N}_j \xi ^{k,N}_j}{\lambda _j}+\frac{\ell ^{5/2}}{\sqrt{2}N^{5/4}}\lambda _j\xi ^{k,N}_j\big (\nabla \varPsi ^N\big (x^{k,N}\big )\big )_j \nonumber \\&\qquad -\frac{\ell ^2}{2N}\big (\xi _j^{k,N}\big )^2 +I_2^N\big (x^{k,N},y^{k,N}\big )+I_3^N\big (x^{k,N},y^{k,N}\big ). \end{aligned}$$

We recall that $I_2^N$ and $I_3^N$ have been defined in Sect. 6. Therefore, using (6.8),

B.2 $$\begin{aligned} Q_{j,\perp }^N= Q^N-Q^N_j=I_1^N+{\tilde{Q}}_j^N, \end{aligned}$$

having set

B.3 $$\begin{aligned} {\tilde{Q}}_j^N&:=-\left( \frac{\ell ^{5/2}}{\sqrt{2}N^{5/4}} -\frac{\ell ^{3/2}}{\sqrt{2}N^{3/4}}\right) \frac{x^{k,N}_j \xi ^{k,N}_j}{\lambda _j} -\frac{\ell ^{5/2}}{\sqrt{2}N^{5/4}}\lambda _j\xi ^{k,N}_j\big (\nabla \varPsi ^N\big (x^{k,N}\big )\big )_j \nonumber \\&\qquad +\frac{\ell ^2}{2N}\big (\xi _j^{k,N}\big )^2. \end{aligned}$$

### Proof of Lemma 6.5

(6.26) is a consequence of the definition (6.24) and the estimate (6.25). Thus, all we have to do is establish the latter. Recalling that $\{{\hat{\phi }}_j\}_{j\in {\mathbb {N}}}:= \{j^{-s}\phi _j\}_{j\in {\mathbb {N}}}$ is an orthonormal basis for ${\mathcal {H}}^s$, we act as in the proof of [17, Lemma 4.7] and obtain

$$\begin{aligned} \left| \left\langle {{\mathbb {E}}_k \varepsilon ^{k,N}},{\hat{\phi }}_j \right\rangle _{s}\right| ^2\lesssim j^{2s}\lambda _j^2{\mathbb {E}}_k \left[ {Q^N_j\big (x^{k,N},\xi ^{k,N}\big )}\right] ^2 \end{aligned}$$

where $Q^N_j$ has been defined in (B.1). Thus

$$\begin{aligned} \left| \left\langle {{\mathbb {E}}_k \varepsilon ^{k,N}},{\hat{\phi }}_j \right\rangle _{s}\right| ^2\lesssim&j^{2s}\lambda _j^2\left( N^{-3/2}{(x^{k,N}_j)^2{\mathbb {E}}_k \xi _j^{2}}\lambda _j^{-2}+N^{-5/2}\lambda _j^2{\mathbb {E}}_k \left[ {\xi _j^2\big (\nabla \varPsi ^N\big (x^{k,N}\big )\big )_j^2}\right] \right) \\&+ j^{2s}\lambda _j^2{\mathbb {E}}_k\big (\left| I_2^N\right| ^2+\left| I_3^N\right| ^2\big )+ j^{2s}\lambda _j^2N^{-2}\\ \lesssim&\, N^{-3/2}{\mathbb {E}}_k \big (j^sx^{k,N}_j\big )^2 +N^{-5/2}j^{-2s}\big (\nabla \varPsi ^N\big (x^{k,N}\big )\big )_j^2\\&+j^{2s}\lambda _j^2N^{-2}+ j^{2s}\lambda _j^2\frac{1+\left| \left| x^{k,N}\right| \right| _{s}^2}{\sqrt{N}}, \end{aligned}$$

where the second inequality follows from the boundedness of the sequence $\{\lambda _j\}$, (6.13) and (6.14). Summing over *j* and applying (2.24) we obtain (6.25). $\square$

### Proof of Lemma 6.6

By definition of $\varepsilon ^{k,N}$, and because $\gamma ^{k,N}=[\gamma ^{k,N}]^2$ (as $\gamma ^{k,N}$ can only take values 0 or 1)

$$\begin{aligned} {\mathbb {E}}_k \left| \left| \varepsilon ^{k,N}\right| \right| _{s}^2= & {} \sum _{j=1}^N j^{2s}\lambda _j^2{\mathbb {E}}_k \left[ \gamma ^{k,N}\left| \xi ^{k,N}_j\right| ^2 \right] \\= & {} \sum _{j=1}^N j^{2s}\lambda _j^2{\mathbb {E}}_k \left[ \left( 1\wedge e^{Q^N\big (x^{k,N},y^{k,N}\big )}\right) \left| \xi ^{k,N}_j\right| ^2\right] . \end{aligned}$$

Using the above, the Lipschitzianity of the function $s \mapsto 1\wedge e^s$, (B.2) and the independence of $Q_{j,\perp }^N$ and $\xi _j^{k,N}$, we write

B.4 $$\begin{aligned}&\left| {\mathbb {E}}_k \left| \left| \varepsilon ^{k,N}\right| \right| _{s}^2 - {\mathrm{Trace}}({\mathcal {C}}_s)\alpha _{\ell }\big (S^{k,N}\big ) \right| \nonumber \\&\quad = \left| {\mathbb {E}}_k \sum _{j=1}^N j^{2s}\lambda _j^2\left( 1\wedge e^{Q^N}\right) \left| \xi _j\right| ^2 -{\mathrm{Trace}}({\mathcal {C}}_s)\alpha _{\ell }\big (S^{k,N}\big ) \right| \nonumber \\&\quad \le \left| {\mathbb {E}}_k \sum _{j=1}^N j^{2s}\lambda _j^2\left( 1\wedge e^{Q^N_{j,\perp }}\right) \left| \xi _j\right| ^2 - {\mathrm{Trace}}({\mathcal {C}}_s)\alpha _{\ell }\big (S^{k,N}\big ) \right| \nonumber \\&\qquad +\left| {\mathbb {E}}_k \sum _{j=1}^N j^{2s}\lambda _j^2 \left[ \left( 1\wedge e^{Q^N}\right) -\left( 1\wedge e^{Q^N_{j,\perp }}\right) \right] \left| \xi _j\right| ^2 \right| \nonumber \\&\quad \lesssim \left| \sum _{j=1}^N j^{2s}\lambda _j^2 {\mathbb {E}}_k\left( 1\wedge e^{Q^N_{j,\perp }}\right) - {\mathrm{Trace}}({\mathcal {C}}_s)\alpha _{\ell }\big (S^{k,N}\big ) \right| \end{aligned}$$

B.5 $$\begin{aligned}&\qquad + \left| {\mathbb {E}}_k \sum _{j=1}^N j^{2s}\lambda _j^2 \left| Q_j^N \right| \left| \xi _j\right| ^2 \right| \end{aligned}$$

We now proceed to bound the addends in (B.4) and (B.5), starting with the latter. Using (B.1) and (B.3), we write

B.6 $$\begin{aligned} {\mathbb {E}}_k \sum _{j=1}^N j^{2s}\lambda _j^2 \left| Q_j^N \right| \left| \xi _j\right| ^2\le&\, {\mathbb {E}}_k \sum _{j=1}^N j^{2s}\lambda _j^2 \left| I_2^N \right| \left| \xi _j\right| ^2+ {\mathbb {E}}_k \sum _{j=1}^N j^{2s}\lambda _j^2 \left| I_3^N \right| \left| \xi _j\right| ^2\nonumber \\&+{\mathbb {E}}_k \sum _{j=1}^N j^{2s}\lambda _j^2 \left| {\tilde{Q}}_j^N \right| \left| \xi _j\right| ^2\nonumber \\ \lesssim&\, \sum _{j=1}^N j^{2s}\lambda _j^2 \sqrt{{\mathbb {E}}_k\left| I_2^N \right| ^2} + {\mathbb {E}}_k \sum _{j=1}^N j^{2s}\lambda _j^2 \sqrt{{\mathbb {E}}_k\left| I_3^N \right| ^2} \nonumber \\&+ \sum _{j=1}^N j^{2s}\lambda _j^2 {\mathbb {E}}_k \left( \left| {\tilde{Q}}_j^N \right| \left| \xi _j\right| ^2\right) \nonumber \\ \lesssim&\,\frac{1+\left| \left| x^{k,N}\right| \right| _{s}}{N^{1/4}}+ \sum _{j=1}^N j^{2s}\lambda _j^2 {\mathbb {E}}_k \left( \left| {\tilde{Q}}_j^N \right| \left| \xi _j\right| ^2\right) , \end{aligned}$$

where the last inequality follows from Lemma 6.4 and (2.16). As for the last addend, using (B.3):

B.7 $$\begin{aligned} \sum _{j=1}^N j^{2s}\lambda _j^2 {\mathbb {E}}_k \left[ \left| {\tilde{Q}}_j^N \right| \left| \xi _j\right| ^2\right] \lesssim&\, \frac{1}{N^{3/4}} \sum _{j=1}^N j^{2s}\lambda _j \left| x^{k,N}_j\right| {\mathbb {E}}_k\left| \xi _j^{k,N} \right| ^3\nonumber \\&+\frac{1}{N^{5/4}} \sum _{j=1}^N j^{2s}\lambda _j^3 \left| \big ({\mathcal {C}}_N\nabla \varPsi ^N\big (x^{k,N}\big )\big )_j\right| {\mathbb {E}}_k\left| \xi _j^{k,N} \right| ^3\nonumber \\&+ \frac{1}{N}\sum _{j=1}^Nj^{2s}\lambda _j^2{\mathbb {E}}_k \left| \xi _j^{k,N} \right| ^4 \nonumber \\ \lesssim&\, \frac{1+ \left| \left| x^{k,N}\right| \right| _{s}^2}{N^{3/4}}, \end{aligned}$$

where the last inequality follows from (2.25), (2.16), the boundedness of the sequence $\{\lambda _j\}_{j\in {\mathbb {N}}}$ and by using Young’s inequality (more precisely, the so-called Young’s inequality “with $\epsilon$”), as follows:

$$\begin{aligned} \lambda _j \left| x^{k,N}_j\right| {\mathbb {E}}_k\left| \xi _j^{k,N} \right| ^3 \le \left| x^{k,N}_j\right| ^2 + \lambda _j^2 \left( {\mathbb {E}}_k\left| \xi _j^{k,N} \right| ^3\right) ^2. \end{aligned}$$

This concludes the analysis of the term (B.5). As for the term (B.4), by definition of $\alpha _{\ell }$, Eq. (1.12),

$$\begin{aligned} 1\wedge e^{Q^{N}_{j,\perp }}-\alpha _{\ell }\big (S^{k,N}\big )&=\left( 1\wedge e^{Q^{N}_{j,\perp }}-1\wedge e^{I^N_1\big (x^{k,N},y^{k,N}\big )}\right) \\&\quad +\left( 1\wedge e^{I^N_1\big (x^{k,N},y^{k,N}\big )}-1 \wedge e^{\ell ^2\big (S^{k,N}-1\big )/2}\right) . \end{aligned}$$

Exploiting the fact that $s\mapsto 1\wedge e^s$ is globally Lipschitz, using Lemma 6.4 and manipulations of the same type as in (B.7), it follows that

$$\begin{aligned} \left| 1\wedge e^{Q^{N}_{j,\perp }}-\alpha _{\ell }\big (S^{k,N}\big )\right| \lesssim \frac{1+S^{k,N}+\left| \left| x^{k,N}\right| \right| _{s}^2}{\sqrt{N}}. \end{aligned}$$

Putting (B.6)–(B.7) and the above together completes the proof. $\square$

## Appendix: Uniform bounds on the moments of $S^{k,N}$ and $x^{k,N}$

### Proof of Lemma 6.2

To prove both bounds, we use a strategy analogous to the one used in [18, Proof of Lemma 9]. Let $\{A_k:k\in {\mathbb {N}}\}$ be any sequence of real numbers. Suppose that there exists a constant $C\ge 0$ (independent of *k*) such that

C.1 $$\begin{aligned} A_{k+1}-A_k\le \frac{C}{\sqrt{N}}\left( 1+A_k\right) . \end{aligned}$$

We start by showing that if the above holds then $A_k\le e^{CT}(A_0+CT)$, uniformly over $k=0,\ldots ,[T\sqrt{N}]$. Indeed, from (C.1),

$$\begin{aligned} A_k\le \left( 1+\frac{C}{\sqrt{N}}\right) ^kA_0+\frac{C}{\sqrt{N}} \sum _{j=0}^{k-1}\left( 1+\frac{C}{\sqrt{N}}\right) ^j \le \left( 1+\frac{C}{\sqrt{N}}\right) ^k\left( A_0+k\frac{C}{\sqrt{N}}\right) . \end{aligned}$$

Thus, for all $k=0,\ldots ,[T\sqrt{N}]$,

$$\begin{aligned} A_k\le \left( 1+\frac{C}{\sqrt{N}}\right) ^{[T\sqrt{N}]}\left( A_0+[T\sqrt{N}] \frac{C}{\sqrt{N}}\right) \le \left( 1+\frac{C}{\sqrt{N}}\right) ^{T\sqrt{N}}(A_0+CT). \end{aligned}$$

Since $1+z\le e^z$ for any $z\in {\mathbb {R}}$,

$$\begin{aligned} \left( 1+\frac{C}{\sqrt{N}}\right) ^{\sqrt{N}}\le \left( e^{C/\sqrt{N}}\right) ^{\sqrt{N}}=e^C. \end{aligned}$$

With this preliminary observation, we can now prove (6.6)–(6.7).

(i) Proof of (6.6). To prove (6.6) we only need to show that (C.1) holds (for some constant $C>0$ independent of *N* and *k*) for the sequence $A_k={\mathbb {E}}_{x^0}{\big (S^{k,N}\big )^q}$. By the definition of $S^{k,N}$, we have
  $$\begin{aligned} S^{k+1,N} = S^{k,N}+\frac{\left| \left| x^{k+1,N}-x^{k,N}\right| \right| _{{\mathcal {C}}_N}^2}{N} +\frac{2\left\langle x^{k+1,N}-x^{k,N},x^{k,N} \right\rangle _{{\mathcal {C}}_N}}{N}. \end{aligned}$$
  Therefore,
  C.2 $$\begin{aligned}&{\mathbb {E}}_{x^0}{(S^{k+1,N})^q}- {\mathbb {E}}_{x^0}\big (S^{k,N}\big )^q \nonumber \\&\quad =\sum _{\begin{array}{c} n+m+l=q \\ (n,m,l)\ne (q,0,0) \end{array}} \left( {\begin{array}{c}q\\ n,m,l\end{array}}\right) {\mathbb {E}}_{x^0}\left[ \big (S^{k,N}\big )^n\left( \frac{\left| \left| x^{k+1,N}-x^{k,N}\right| \right| _{{\mathcal {C}}_N}^2}{N}\right) ^m\right. \nonumber \\&\qquad \left. \times \left( \frac{2\left\langle x^{k+1,N}-x^{k,N},x^{k,N} \right\rangle _{{\mathcal {C}}_N}}{N}\right) ^l\right] . \end{aligned}$$
  Thus, to establish (C.1) it is enough to argue that each of the terms in the right-hand side of the above is bounded by $(C/\sqrt{N})(1+{\mathbb {E}}{\big (S^{k,N}\big )^q})$. To this end, set
  $$\begin{aligned} J^{k,N}&:= {\mathbb {E}}_{x^0}\left[ {\big (S^{k,N}\big )^n\left( \frac{\left| \left| x^{k+1,N}-x^{k,N}\right| \right| _{{\mathcal {C}}_N}^2}{N}\right) ^m \left( \frac{2\left\langle x^{k+1,N}-x^{k,N},x^{k,N} \right\rangle _{{\mathcal {C}}_N}}{N}\right) ^l} \right] \\&= {\mathbb {E}}_{x^0}{\mathbb {E}}_k \left[ {\big (S^{k,N}\big )^n\left( \frac{\left| \left| x^{k+1,N} -x^{k,N}\right| \right| _{{\mathcal {C}}_N}^2}{N}\right) ^m\left( \frac{2\left\langle x^{k+1,N}-x^{k,N},x^{k,N} \right\rangle _{{\mathcal {C}}_N}}{N}\right) ^l} \right] . \end{aligned}$$
  By the Cauchy–Schwartz inequality for the scalar product $\left\langle \cdot ,\cdot \right\rangle _{{\mathcal {C}}_N}$,
  $$\begin{aligned} \frac{\left\langle x^{k+1,N}-x^{k,N},x^{k,N} \right\rangle _{{\mathcal {C}}_N}^l}{N^l}&\le \frac{\left| \left| x^{k,N}\right| \right| _{{\mathcal {C}}_N}^l\left| \left| x^{k+1,N}-x^{k,N}\right| \right| _{{\mathcal {C}}_N}^l}{N^l}\\&=\big (S^{k,N}\big )^{l/2}\frac{\left| \left| x^{k+1,N}-x^{k,N}\right| \right| _{{\mathcal {C}}_N}^l}{N^{l/2}}, \end{aligned}$$
  which gives
  $$\begin{aligned} J_k^N\lesssim {\mathbb {E}}_{x^0}\left[ \big (S^{k,N}\big )^{n+l/2}\frac{{\mathbb {E}}_k \left| \left| x^{k+1,N} -x^{k,N}\right| \right| _{{\mathcal {C}}_N}^{2m+l}}{N^{m+l/2}}\right] . \end{aligned}$$
  Using the bound (6.4) of Lemma 6.1, we also have
  $$\begin{aligned} {\mathbb {E}}_k\frac{{\left| \left| x^{k+1,N}-x^{k,N}\right| \right| _{{\mathcal {C}}_N}^{2m+l}}}{N^{m+l/2}}\lesssim \frac{\big (S^{k,N}\big )^{m+l/2}}{N^{m+l/2}}+\frac{1}{N^{(m+l/2)/2}}. \end{aligned}$$
  Putting all of the above together (and using Young’s inequality) we obtain
  $$\begin{aligned} J_k^N \lesssim \frac{{\mathbb {E}}_{x^0}[\big (S^{k,N}\big )^q]}{N^{m+l/2}}+ \frac{1}{N^{(m+l/2)/2}}. \end{aligned}$$
  Now observe that $(m+l/2)/2\ge 1/2$ except when $(n,m,l)=(q,0,0)$ or $(n,m,l)=(q-1,0,1)$. Therefore we have shown the desired bound for all the terms in the expansion (C.2), except the one with $(n,m,l)=(q-1,0,1)$. To study the latter term, we recall that $\gamma ^{k,N}\in \{0,1\}$, and use the definition of the chain [Eqs. (2.8) and (2.12)] to obtain
  $$\begin{aligned} \left| \left\langle x^{k+1,N}-x^{k,N},x^{k,N} \right\rangle _{{\mathcal {C}}_N}\right| \lesssim&\, \delta \left| \left| x^{k,N}\right| \right| _{{\mathcal {C}}_N}^2+ \delta \left| \left\langle {\mathcal {C}}_N\nabla \varPsi ^N\big (x^{k,N}\big ),x^{k,N} \right\rangle _{{\mathcal {C}}_N}\right| \\&+ \sqrt{\delta }\left| \left\langle x^{k,N},({\mathcal {C}}_N)^{1/2}\xi ^{k,N} \right\rangle _{{\mathcal {C}}_N}\right| . \end{aligned}$$
  Combining (2.26) with the Cauchy–Schwartz inequality we have
  $$\begin{aligned} \delta \left| \left\langle {\mathcal {C}}_N\nabla \varPsi ^N\big (x^{k,N}\big ),x^{k,N} \right\rangle _{{\mathcal {C}}_N}\right| \lesssim N^{-1/2}\left( 1+\left| \left| x^{k,N}\right| \right| _{{\mathcal {C}}_N}^2\right) \lesssim N^{-1/2}+N^{1/2}S^{k,N}, \end{aligned}$$
  where in the last inequality we used the following observation
  $$\begin{aligned} \left| \left| x^{k,N}\right| \right| _{s}^2=\sum _{j=1}^\infty \big (x^{k,N}\big )_j^2j^{2s}=\sum _{j=1}^\infty \frac{\big (x^{k,N}\big )_j^2}{\lambda _j^2}(\lambda _j^2j^{2s})\lesssim \sum _{j=1}^\infty \frac{\big (x^{k,N}\big )_j^2}{\lambda _j^2}=NS^{k,N}. \end{aligned}$$
  Recalling that $\left\langle x^{k,N},({\mathcal {C}}_N)^{1/2}\xi ^{k,N} \right\rangle _{{\mathcal {C}}_N}$, conditioned on $x^{k,N}$, is a linear combination of zero-mean Gaussian random variables, we have
  $$\begin{aligned} {\mathbb {E}}_k \sqrt{\delta }\left| \left\langle x^{k,N},({\mathcal {C}}_N)^{1/2}\xi ^{k,N} \right\rangle _{{\mathcal {C}}_N}\right|&\lesssim 1+N^{-1/2}{\mathbb {E}}_k \left| \left\langle x^{k,N},({\mathcal {C}}_N)^{1/2}\xi ^{k,N} \right\rangle _{{\mathcal {C}}_N}\right| ^2\\&\lesssim 1+\sqrt{N}S^{k,N}. \end{aligned}$$
  Putting the above together and taking expectations we can then conclude
  $$\begin{aligned} {\mathbb {E}}\left[ \frac{\big (S^{k,N}\big )^{q-1}\left\langle x^{k+1,N}-x^{k,N},x^{k,N} \right\rangle _{{\mathcal {C}}_N}}{N} \right]&\lesssim \frac{{\mathbb {E}}\left[ \big (S^{k,N}\big )^{q-1} \right] }{N}+\frac{{\mathbb {E}}\left[ \big (S^{k,N}\big )^q \right] }{\sqrt{N}}\\&\lesssim (1/\sqrt{N})\big (1+{\mathbb {E}}\left[ \big (S^{k,N}\big )^q \right] \big ), \end{aligned}$$
  and (6.6) follows.
(ii) Proof of (6.7). This is very similar to the proof of (6.6), so we only sketch it. Just as before, it is enough to establish the following bound
  $$\begin{aligned}&{\mathbb {E}}\left[ \left| \left| x^{k,N}\right| \right| _{s}^{2n}\left| \left| x^{k+1,N}-x^{k,N}\right| \right| _{s}^{2m}\left\langle x^{k+1,N}-x^{k,N},x^{k,N} \right\rangle _{s}^{l} \right] \\&\quad \lesssim \frac{1}{\sqrt{N}}\left( 1+{\mathbb {E}}\left[ \left| \left| x^{k,N}\right| \right| _{s}^{2q} \right] \right) \end{aligned}$$
  for each (*n*, *m*, *l*) such that $n+m+l=q$ with the exception of the triple $(n,m,l)=(q,0,0)$. Applying the Cauchy–Schwartz inequality for $\left\langle \cdot ,\cdot \right\rangle _{s}$ we have
  $$\begin{aligned} \left\langle x^{k+1,N}-x^{k,N},x^{k,N} \right\rangle _{s}^{l}\le \left| \left| x^{k,N}\right| \right| _{s}^l\left| \left| x^{k+1,N}-x^{k,N}\right| \right| _{s}^l. \end{aligned}$$
  Thus, Lemma 6.1 implies
  $$\begin{aligned}&{\mathbb {E}}_k \left| \left| x^{k,N}\right| \right| _{s}^{2n}\left| \left| x^{k+1,N}-x^{k,N}\right| \right| _{s}^{2m}\left\langle x^{k+1,N}-x^{k,N},x^{k,N} \right\rangle _{s}^{l}\\&\quad \le \left| \left| x^{k,N}\right| \right| _{s}^{2n+l}{\mathbb {E}}_k \left| \left| x^{k+1,N}-x^{k,N}\right| \right| _{s}^{2m+l} \\&\quad \lesssim \frac{\left| \left| x^{k,N}\right| \right| _{s}^{2n+l}(1+\left| \left| x^{k,N}\right| \right| _{s}^{2m+l})}{N^{(m+l/2)/2}}. \end{aligned}$$
  The above gives us the desired bound for all (*n*, *m*, *l*) except for $(n,m,l)=(q-1,0,1)$. Like before, to study the latter case we observe
  $$\begin{aligned}&\left\langle x^{k+1,N}-x^{k,N},x^{k,N} \right\rangle _{s}\\&\quad =\,\gamma ^{k,N}\left( -\frac{\ell }{\sqrt{N}} \left( \left| \left| x^{k,N}\right| \right| _{s}^2+\left\langle {\mathcal {C}}_N\nabla \varPsi ^N\big (x^{k,N}\big ),x^{k,N} \right\rangle _{s}\right) \right. \\&\qquad \left. +\frac{\sqrt{2\ell }}{N^{1/4}}\left\langle ({\mathcal {C}}_N)^{1/2}\xi ^{k,N},x^{k,N} \right\rangle _{s}\right) \\&\quad \lesssim \frac{1}{\sqrt{N}}\left( 1+\left| \left| x^{k,N}\right| \right| _{s}^2\right) +\frac{1}{N^{1/4}}\gamma ^{k,N}\left\langle (C_N)^{1/2}\xi ^{k,N},x^{k,N} \right\rangle _{s}\\&\quad \lesssim \frac{1}{\sqrt{N}}\left( 1+\left| \left| x^{k,N}\right| \right| _{s}^2\right) , \end{aligned}$$
  where penultimate inequality follows from the Cauchy–Schwartz inequality, (2.25), and the fact that $\gamma ^{k,N}\in \{0,1\}$, and the last inequality follows from Lemma 6.5. This concludes the proof. $\square$

### Remark C.1

In [17] the authors derived the diffusion limit for the chain under weaker assumptions on the potential $\varPsi$ than those we use in this paper. Essentially, they assume that $\varPsi$ is quadratically bounded, while we assume that it is linearly bounded. If $\varPsi$ was quadratically bounded the proof of Lemma 6.5 would become considerably more involved. We observe explicitly that the statement of Lemma 6.5 is of paramount importance in order to establish the uniform bound on the moments of the chain $x^{k}$ contained in Lemma 6.2. In [17] obtaining such bounds is not an issue, since the authors study the chain in its stationary regime. In other words, in [17] the law of $x^{k,N}$ is independent of *k*, and thus the uniform bounds on the moments of $x^{k,N}$ and $S^{k,N}$ are automatically true for target measures of the form considered there (see also the first bullet point of Remark 4.1). $\square$

## Footnotes

1 In this paper, we commit a slight abuse of our notation by writing ${\mathcal {C}}_s$ to mean the covariance operator on the Sobolev-like subspace ${\mathcal {H}}^s$ and ${\mathcal {C}}_N$ to mean that on the finite dimensional subspace $X^N$ as defined in (1.5). We distinguish these two by always employing *N* as the subscript for the latter, and lower case letters such as *s* or *r* for the former.

2 Notice that $S^{k,N}$ is only a function of $x^{k,N}$.

3 Note that in the limit the dependence of the drift on $S^{k,N}$ becomes explicit.
